# Probing the Energy Landscape of α‐Synuclein Amyloid Fibril Formation by Systematic K‐to‐Q Mutagenesis

**DOI:** 10.1002/advs.77643

**Published:** 2026-09-27

**Authors:** Antonin Kunka, Azad Farzadfard, Jacob Aunstrup Larsen, Rasmus Krogh Norrild, Federica Saraceno, Celia Fricke, Hossein Mohammad‐Beigi, Ahmed Sadek, Jonas Folke, Susana Aznar, Alexander K. Buell

**Affiliations:** ^1^ Department of Biotechnology and Biomedicine Technical University of Denmark Lyngby Denmark; ^2^ Laboratory of Protein Design and Immunoengineering Institute of Bioengineering, School of Life Sciences, École Polytechnique Fédérale de Lausanne Lausanne Switzerland; ^3^ Centre For Neuroscience and Stereology Department of Neurology Copenhagen University Hospital Bispebjerg and Frederiksberg Hospital Copenhagen Denmark

**Keywords:** Energy Landscape, IDPs, Mutagenesis, Parkinson's Disease, Polymorph

## Abstract

Aggregation of α‐synuclein (αSyn) is a hallmark of Parkinson's disease, yet its pathological roles remain poorly understood. Elucidating these roles requires detailed knowledge of the free energy landscape of αSyn self‐assembly, i.e. the states that can be populated and their mechanisms and rates of interconversion. Here, we quantitatively probe this landscape using systematic mutational analysis, focusing on electrostatic contributions. We designed and produced 41 αSyn variants containing one to six acetyllysine‐mimicking (K‐Q) mutations to dissect the effects on key assembly pathways, fibril stability, and polymorphism. We derived a quantitative framework for the analysis of mutational effects and used it to isolate general electrostatic from residue‐specific effects on *de novo* αSyn aggregation. We established fibril stability and relative growth rate analysis as indirect biophysical probes of fibril polymorphism and identified K43Q+K45Q and K80Q as mutations with the most significant effect onamyloid formation. Moreover, we demonstrate that a subset of variants, most noticeably K58Q+K60Q, show differential sensitivity toward disease‐derived fibril polymorphs in seed amplification assays. Clustering analysis reveals that the mutational effects map onto the structural context of the lysine residues in known αSyn fibril structures. Together, this work provides a scalable, quantitative framework for probing the complex αSyn assembly landscape using mutational analysis.

## Graphical abstract

We use lysine‐to‐glutamine mutations to study the effect of electrostatics on the kinetics and thermodynamics of alpha‐synuclein amyloid fibril formation. We find that mutational effects map on their structural context within fibrils and identify residues that modulate the energy landscape of alpha‐synuclein self‐assembly. Our work outlines a scalable, quantitative framework that increases the mechanistic insight of mutational studies of αlpha‐synuclein amyloid formation.

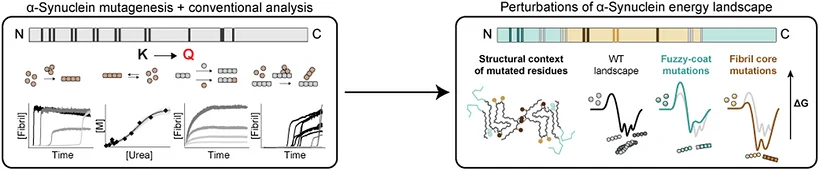

## 1 Introduction

α‐Synuclein (αSyn) is a 14.5 kDa protein present in neurons, the peripheral nervous system and red blood cells [1, 2, 3]. It is involved in synaptic vesicle trafficking, dopamine regulation, calcium signalling, mitochondrial function, and lipid metabolism [4, 5, 6, 7, 8, 9]. Pathologically, αSyn is the principal component of intracellular inclusions that characterize synucleinopathies, including Parkinson's disease (PD), Lewy body dementia (LBD), and multiple system atrophy (MSA) [10]. Although it has been demonstrated that αSyn aggregates are toxic and propagate between cells in a prion‐like manner, it remains unclear whether disease arises from gain‐of‐function toxicity or loss of protein function [11, 12, 13, 14, 15].

One major obstacle to resolving αSyn's role in disease is the complexity of the protein itself. Even outside of its biological background, characterization of αSyn presents a multifaceted challenge due to its conformational plasticity and context‐dependent behaviour. Its ability to transition from a disordered monomeric state to various oligomeric or fibrillar forms, each potentially associated with distinct cellular outcomes, complicates both the mechanistic understanding of the associated diseases and their therapeutic targeting [13, 15, 16, 17]. This structural flexibility is also modulated by mutations, post‐translational modifications (PTMs), differential expression, and cellular localization, further complicating the distinction between physiological and pathological states [18, 19, 20, 21, 22, 23, 24].

The complex energy landscape of αSyn is often probed through a combination of advanced biophysical methods (e.g., NMR or single molecule FRET), which provide valuable high‐resolution information but are often not scalable [25, 26, 27, 28, 29]. A compelling approach is to instead exploit mutational analysis which is well established for studying the energetics and mechanisms of folding and binding of globular proteins [30, 31, 32, 33]. Studies involving familiar mutants [34, 35], N‐ and C‐ terminal truncations [36, 37, 38], PTMs and their mimetics [39, 40, 41, 42, 43], and large‐scale variant analyses [44, 45] have collectively highlighted regions critical for αSyn aggregation, suggesting that mutational scanning can indirectly map features of the underlying energy landscape, analogous to what is possible for folded proteins. Despite the wealth of insightful results from these and other studies [46, 47, 48, 49, 50, 51, 52, 53], our ability to predict how individual substitutions alter specific assembly pathways of αSyn (e.g., nucleation, elongation, seed amplification) within specific biological contexts remains limited.

Mutational studies of αSyn generally investigate three classes of protein variants: (i) physiologically or pathologically relevant variants (e.g., disease‐associated familial mutants), (ii) targeted sequence perturbations designed to test specific hypotheses or probe mechanisms (e.g., Φ‐value analysis) [54], and (iii) random variants used for unbiased searches that typically require large libraries to be informative. The first class can provide direct insight into pathogenic mechanisms by revealing how disease mutations reshape the energy landscape and assembly behaviour. However, their number is generally too low to provide global insights into the molecular principles governing αSyn assembly across sequence space. The second approach enables detailed physico‐chemical and mechanistic interpretation through comparison with the WT protein. However, such analyses are only valid if the introduced mutations do not substantially distort the energy landscape or introduce alternative folding or assembly pathways. Consequently, it is essential to characterize the accessible structural states of each variant and interpret mechanistic differences with caution when mutagenesis substantially remodels the underlying energy landscape. Random variant libraries often contain a large proportion of uninformative mutants, and the observed effects are frequently confounded by the properties of the screening or selection assay.

In this study, we adopt a hybrid approach by systematically investigating a large panel of 41 acetyllysine‐mimicking variants. Lysine acetylation is a particularly attractive perturbation because it directly alters the electrostatic properties of αSyn while remaining biologically relevant. Acetylated lysine residues have been identified in vivo in soluble αSyn monomers [55] as well as in fibril structures isolated from the brains of patients with MSA [56]. By leveraging a large set of disease‐relevant perturbations, we ask to what extent systematic mutational analysis coupled with scalable bulk biophysical assays can provide quantitative insight into how electrostatic interactions shape the αSyn assembly energy landscape. By constraining the aggregation conditions to favour a limited set of microscopic pathways, we aimed to assess how these sequence changes reshape the energy landscape of αSyn under in vitro conditions reflecting specific biological contexts. We observed that mutational effects fall broadly into two categories: those that affect the energy barriers on a WT‐like landscape, and those that reshape the energy landscape and create minima not easily accessible by WT under the same solution conditions. Our work establishes a scalable, quantitative framework that increases the informational output of mutational studies of αSyn using detailed biophysical assays, and providing deep insights into its energy landscape of amyloid formation.

## 2 Results

### 2.1 Design, Overview and Naming Convention of αSyn Mutants Used in this Study

**FIGURE 1:**
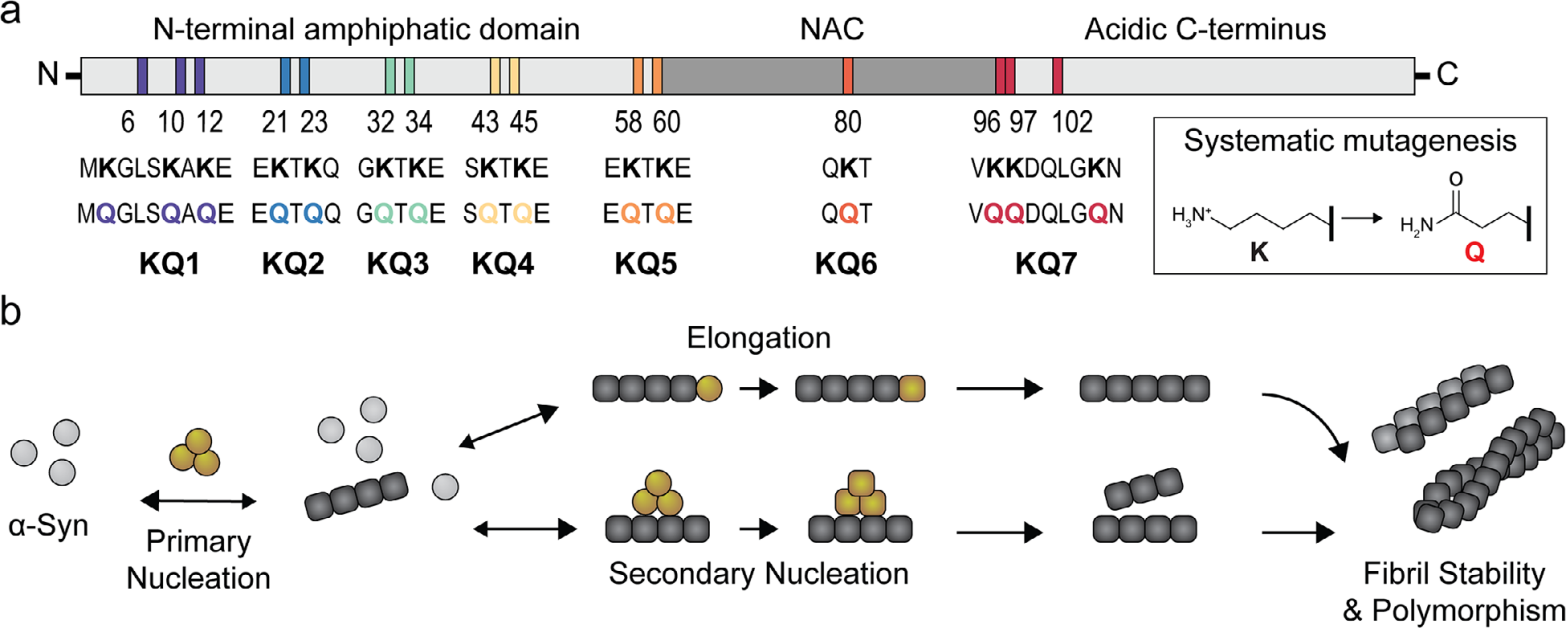
Overview of the αSyn variants and aggregation pathways involved in this study. (a) Schematic overview of the αSyn sequence with highlighted positions of lysine residues mutated to glutamines in this study. The αSyn variants containing 2–3 mutations of adjacent lysines are referred to as KQ1‐7 cluster mutants based on their position in the sequence from N to C terminus (e.g., KQ1 = K6Q + K10Q + K12Q, KQ2 = K21Q + K23Q, etc., Table s1). b) Schematic view of the distinct aggregation pathways and aggregate properties of αSyn probed in this study.

αSyn contains 15 lysine residues distributed across the sequence, grouped in clusters containing 1 to 3 lysine residues within imperfect repeats (Figure 1a). We substituted lysines with glutamines as an amino‐acid with similar size to lysine with physiochemical properties mimicking lysine acetylation as a physiologically relevant posttranslational modification [43, 56, 57]. We generated 41 variants including all 15 single‐point K‐to‐Q mutants, six mutant cluster variants (two 3‐point mutants: KQ1 and KQ7; four 2‐point mutants: KQ2, KQ3, KQ4, and KQ5), and their combinations (Figure 1a; Table S1 and Tables 1, 2, 3). We primarily focused on the KQ cluster variants (i.e., numbered according to their position in the sequence from N to C terminus, with KQ6 designating the single point mutation K80Q for completeness) as reporters of the sequence dependency of this particular type of mutation. The rest of the variants were used to support and extend the observations made with the KQ cluster variants, e.g., discern contributions of individual mutations within each KQ cluster, or study epistatic effects between the clusters in aggregation assays (Figure 1b; Figure S1).

**TABLE 1:** List of primers used for mutagenesis. The random flanking sequence upstream of the BsaI recognition site (GGTCTC), an extra base next to which BsaI cleaves, and mutated nucleotides are shown in lowercase. Reverse (R) and forward (F) direction of primer is encoded in the primer name.

Primer n.	Primer name	Mutations	Sequence (5'‐3')
1	αSyn _F_8‐15	—	ggctacGGTCTCaTGTCAAAAGCCAAGGAAGGAGTGG
2	αSyn _R_K6Q	K6Q	ggctacGGTCTCaGACAGACCTTgCATGAAGACGTCCATATGT
3	αSyn _R_8‐15	—	ggctacGGTCTCaGACAGACCTTTCATGAAGACGTCC
4	αSyn _F_K10Q	K10Q	ggctacGGTCTCaTGTCAcAAGCCAAGGAAGGAGTGGTGG
5	αSyn _F_K12Q	K12Q	ggctacGGTCTCaTGTCAAAAGCCcAGGAAGGAGTGGTGG
6	αSyn _F_K10Q+K12Q	K10Q+K12Q	ggctacGGTCTCaTGTCAcAAGCCcAGGAAGGAGTGGTGGCAGC
7	αSyn _F_26‐31	—	ggctacGGTCTCaTTGCGGAAGCAGCGGG
8	αSyn _R_K21Q	K21Q	ggctacGGTCTCaGCAACACCCTGTTTGGTCTgTTCCGC
9	αSyn _R_K23Q	K23Q	ggctacGGTCTCaGCAACACCCTGTTgGGTCTTTTCCGC
10	αSyn _R_K21Q+K23Q	K21Q+K23Q	ggctacGGTCTCaGCAACACCCTGTTgGGTCTgTTCCGCG
11	αSyn _F_39‐46	—	ggctacGGTCTCaGTACGTAGGTTCGAAGACGAAGGA
12	αSyn _R_K32Q	K32Q	ggctacGGTCTCaGTACAAAACTCCCTCTTTTGTTTgCCCCGC
13	αSyn _R_K34Q	K34Q	ggctacGGTCTCaGTACAAAACTCCCTCTTgTGTTTTCCCCGC
14	αSyn _R_K32Q+K34Q	K32+K34Q	ggctacGGTCTCaGTACAAAACTCCCTCTTgTGTTTgCCCCGCT
15	αSyn _R_30‐39	—	ggctacGGTCTCaGTACAAAACTCCCTCTTTTGTTTTCCCC
16	αSyn _F_K43Q	K43Q	ggctacGGTCTCaGTACGTAGGTTCGcAGACGAAGGAAGGC
17	αSyn _F_K45Q	K45Q	ggctacGGTCTCaGTACGTAGGTTCGAAGACGcAGGAAGGC
18	αSyn _F_K43Q+K45Q	K43+K45Q	ggctacGGTCTCaGTACGTAGGTTCGcAGACGcAGGAAGGCGT
19	αSyn _R_63‐69	—	ggctacGGTCTCaTCACAAATGTGGGTGGAGCTG
20	αSyn _R_K58Q	K58Q	ggctacGGTCTCaGTGACTTGCTCTTTTGTCTgTTCTGCTACG
21	αSyn _R_K60Q	K60Q	ggctacGGTCTCaGTGACTTGCTCTTgTGTCTTTTCTGCTACG
22	αSyn _R_K58Q+K60Q	K58Q+K60Q	ggctacGGTCTCaGTGACTTGCTCTTgTGTCTgTTCTGCTACGG
23	αSyn _F_85‐89	—	ggctacGGTCTCaCGCGGGCTCAATTGCTG
24	αSyn _R_K80Q	K80Q	ggctacGGTCTCaCGCGCCCTCTACAGTCTgTTGCG
25	αSyn _F_99‐105	—	ggctacGGTCTCaCAGCTTGGCAAGAACGAAGAGG
26	αSyn _R_K96Q	K96Q	ggctacGGTCTCaGCTGGTCTTTCTgGACGAATCCGG
27	αSyn _R_K97Q	K97Q	ggctacGGTCTCaGCTGGTCTTgCTTGACGAATCCGG
28	αSyn _R_K96Q+K97Q	K96Q+K97Q	ggctacGGTCTCaGCTGGTCTTgCTgGACGAATCCGGTCG
29	αSyn _R_93‐100	—	ggctacGGTCTCaGCTGGTCTTTCTTGACGAATCCG
30	αSyn _F_K102Q	K102Q	ggctacGGTCTCaCAGCTTGGCcAGAACGAAGAGGGCG

**TABLE 2:** Composition of the reaction mixtures for mutagenesis (PCR 1) and Golden Gate Assembly (PCR 2). Phusion MM – master mix containing HF‐Phusion DNA Polymerase, dNTPs, and buffer components.

PCR 1 – Amplification mixture			PCR 2 –Golden gate assembly		
Component	Stock	Final	Component	Stock	Final
2x Phusion MM	2x	1x	T4 buffer	10x	1x
Fwd_primer	10 µM	0.5 µM	T4 ligase	400 kU/mL	400 U/mL
Rev_primer	10 µM	0.5 µM	BsaI‐HF	20 kU/mL	1.2 kU/mL
Plasmid DNA	50‐200 ng/µL	100 ng	PCR mix	50‐200 ng/µL	100 ng
mqH_2_O	—	—	mqH_2_O	—	—

**TABLE 3:** Thermocycler protocol used for the PCR 1 mutagenesis. Times and temperatures were adjusted based on the size of the plasmid (approx. 5.8 kb) and fidelity of the HF‐Phusion polymerase.

Step	Temperature (°C)	Time (s)	n. cycles
Initial denaturation	98	30	1
Denaturation	98	5	28
Annealing	55	15	
Extension	72	180	
Final Extension	72	600	1

### 2.2 Dissecting Global and Sequence‐Specific Effects of KQ Mutations on αSyn Aggregation

**FIGURE 2:**
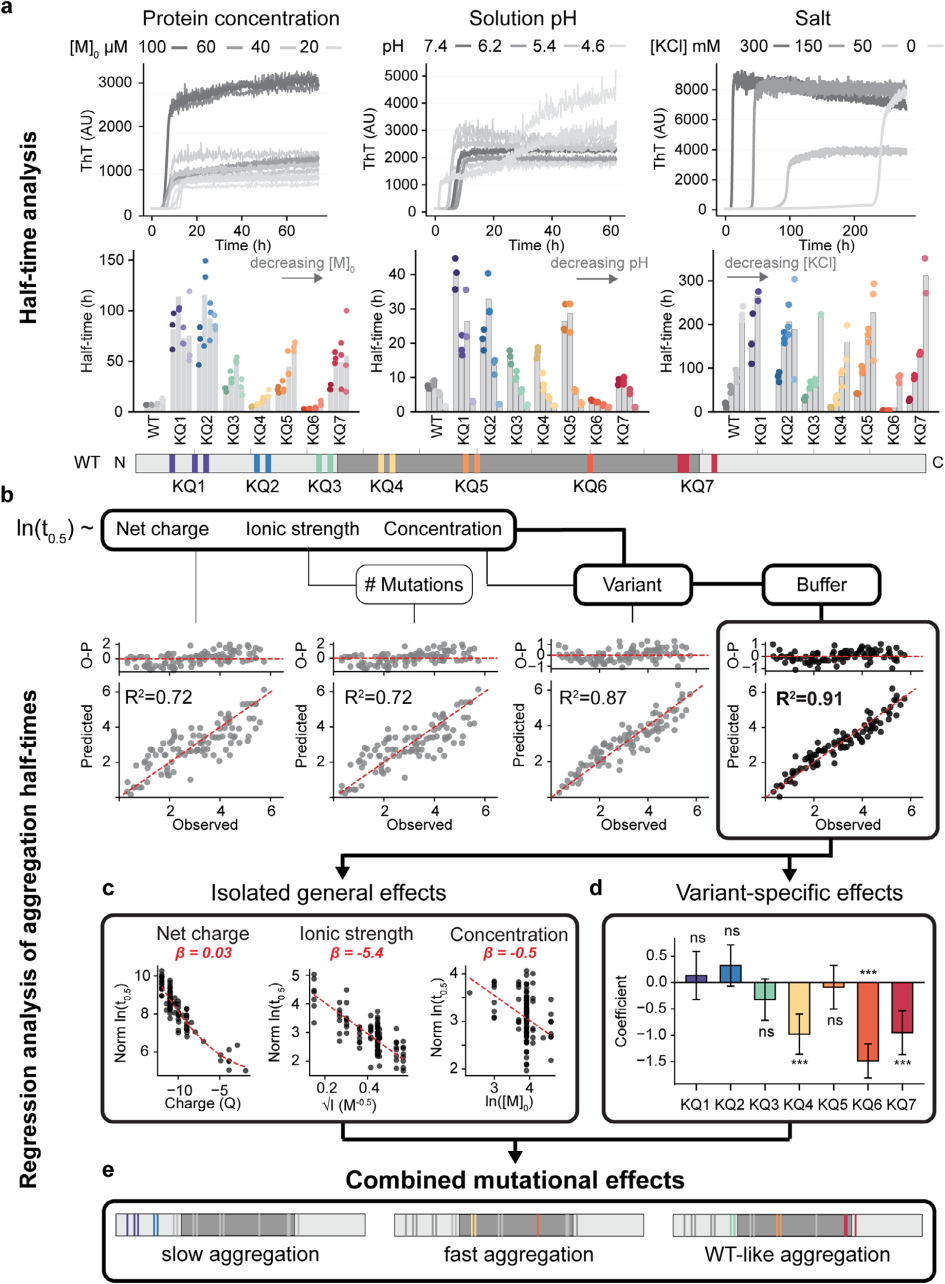
The effects of KQ mutations on *de novo* aggregation of αSyn. (a) Half‐time analysis of ThT kinetics. Top: Aggregation kinetics of wild type αSyn monitored with ThT in 96‐well plates with continuous shaking and varying initial monomer concentration (left), buffer pH (middle), or salt concentration (right). Bottom: Aggregation half‐times extracted from aggregation kinetics of WT and KQ cluster mutants shown in (a) and Figure S2 by fitting with Equation (M1). The circles correspond to values from individual experiments, bars correspond to the means (n = 3). WT and KQ clusters are color‐coded in grayscale and hues indicated in the sequence scheme below, respectively. The order of the bars in each plot corresponds to the descending values of the variable parameters (i.e., protein concentration 100–20 µM, pH 7.4‐4.6, and [KCl] 300‐0 mM) highlighted by a color‐intensity gradient as illustrated for WT in the top graph's legend. The dark and light areas in the sequence scheme correspond to regions found in the fibril cores and their fuzzy coats, respectively. b) Regression analysis of aggregation half‐times. The mean values of the half‐times (corresponding to the bar plots in (a) were log‐transformed, weighted by their respective SD (w = mean/SD), parameterized for each experimental condition, and modelled using different combinations of global scaling (charge dependency ∼ Q^2^, ionic strength ∼√I, protein concentration ∼ ln[M]_0_, grey box) and additional parameters. The four scatter plots show correlations between experimentally measured (observed) and predicted values from models involving (left) global scaling alone, (middle‐left) global scaling + number of mutations, (middle‐right) global scaling + variant specific coefficients, and (right) global scaling + variant specific coefficients + experimental conditions (i.e. buffer type: phosphate, citrate, tris). The red line corresponds to the line of slope 1, with the residuals showing the difference between observed and predicted values. (c) Decomposition of global and (d) variant‐specific scaling coefficients from the best model based on the explained variance (top right). The model prediction (red line) is overlayed with the observed data (black circles) normalized for all but one model parameter (e.g., for the left graph depicting net charge scaling: norm ln(t_0.5_) = ln(t_0.5_)—β_c_*ln[M]_0_ – β_I_*√I—β_v_*Variant—β_b_*buff). Positive values of variant specific coefficients (β_v_, d) indicate that the variants are on average slower compared to the global model predictions, whereas variants with negative intercepts are faster than predicted based on global effects alone. Error bars indicate ± 95% confidence intervals. Asterisks denote levels of statistical significance: *p < 0.05; **p < 0.01; ***p < 0.001, ns – not significant. Parameters from all models are provided in Table S2. (e) Classification of KQ cluster variants according to their combined effects on aggregation.

To dissect the effects of positive charge removal on αSyn aggregation, we measured the aggregation kinetics of WT and KQ cluster variants under a range of carefully selected solution conditions, including varying pH, monomer and salt concentrations using a Thioflavin‐T (ThT) fluorescence assay (Figure 2a; Figure S2). In line with previous reports, we observed that lowering the pH and increasing the salt concentration promoted aggregation overall, whereas variation of the initial monomer concentration had only a moderate effect [38, 58].

Comparison of the extracted aggregation half‐times (Equation M1, 59) as a crude measure for overall aggregation rates revealed that variants with lysine mutations in the N‐terminus (KQ1, KQ2) showed generally slower aggregation compared to the WT (Figure 2a). In contrast, mutation of residue K80 found in most fibril cores (variant KQ6) accelerated the aggregation under most of the conditions. The observed differences between the aggregation behaviour of the WT and other variants depended on the experimental conditions, which complicated drawing general conclusions about their effects (Figure 2a).

To avoid the cumbersome direct comparison of mutational effects across each of the twelve conditions (4 in each of the 3 assays), we used weighted linear regression analysis as a framework to isolate general effects from sequence‐specific and other contributions to αSyn aggregation. The aggregation half‐times obtained from fitting individual kinetic curves were averaged across triplicates, yielding a comprehensive dataset of 96 mean values ± SDs (File S1). We parameterized each datapoint by variables describing both extrinsic factors (buffer type, salt type, ionic strength, pH) and intrinsic sequence features (number of mutations, variant identity, and theoretical net charge calculated using Equation M2). The mutations and assay conditions were designed to create a parameter space suitable for deconvolving electrostatic from other energetic contributions. Our modelling framework is comprised of a combination of general (G), sequence‐specific (S), and experimental condition (E) terms according to Equations ((1), (2), (3), (4)).

(1) $$\ln\left(t_{0.5}\right)=G+S+E+\varepsilon$$

(2) $$G=\beta_{0}+\beta_{c}\ln{\left[M\right]}_{0}+\beta_{Q}Q^{2}+\beta_{I}\sqrt{I}$$

(3) $$S=\sum \beta_{v}var+\beta_{m}\#muts$$

(4) $$E=\beta_{b}buff$$

where ε is the residual error, β_0_ is the intercept; *[M]_0_*, *Q*, and *I* denote initial monomer concentration, theoretical net charge, and ionic strength, with coefficients β_c_, β_Q_, and β_I_, respectively; #muts is the number of mutations with coefficient β_m_; and *var* and *buff* are binary indicators for variant and buffer condition, with coefficients β_v_ and β_b_.

The general scaling term (G, Equation 2) was modelled based on the following assumptions: (i) Under a nucleation–polymerization mechanism, the log‐transformed aggregation half‐time ln(t_0.5_) scales linearly with the logarithm of the initial monomer concentration (ln[M]_0_) [60]. (ii) Electrostatic interactions can be described by the Debye–Hückel theory, according to which the interaction strength scales with the square of the net charge (Q^2^) and is exponentially attenuated with the square root of the solution ionic strength (√I).

The framework defined by Equations ((1), (2), (3), (4)) allowed us to test different hypotheses and compare models with different numbers of free parameters, using the Bayesian Information Criterion (BIC) and the Akaike Information Criterion (AIC) to evaluate goodness of fit while penalizing model complexity (Table S2). First, we investigated how much of the experimentally observed variation can be explained by simple scaling of general parameters (Equation 2). The adjusted R^2^ of the resulting model was 0.72 (Figure 2b, left) and did not improve when the number of mutations was considered (i.e., Equation 1 with E = 0, ∑β_v_ = 0, Figure 2b, middle‐left). In contrast, using variant‐specific coefficients instead of the number of mutations significantly improved the fit (Equation 1 with E = 0, β_m_ = 0, adjusted R^2^ = 0.87, Figure 2b, middle‐right). We observed that the half‐time values from the pH screening assay are lower compared to those from other assays under comparable conditions (pH 7.4, ionic strength and protein concentration). This suggests the presence of specific interactions of αSyn with the citrate buffer (used in the pH screening assay) beyond simple charge screening, analogous to the effects of bivalent ions on αSyn aggregation observed previously [61]. Indeed, adding a buffer‐specific coefficient improved R^2^ with statistical significance, yielding the final model described by Equation (1) with β_m_ = 0 that could explain most of the variance (91%) in the data (Table S2 and Figure S3).

The model coefficients for the general terms are consistent with the expected scaling behavior (Figure 2c; Table S2). Aggregation exhibits a moderate dependence on protein concentration (β_c_ ≈ −0.5), a pronounced screening effect from ionic strength (β_I_ ≈ −5), and a quadratic scaling with net charge (β_Q_ ≈ 0.03) (Figure 2c). The scaling with protein concentration is consistent with weakly monomer‐dependent (saturated) elongation and secondary processes (e.g., fragmentation, or saturated secondary nucleation) as dominant aggregate growth and proliferation processes under these conditions (continuous shaking with a bead in the well), as previously described for WT αSyn [38, 62, 63, 64, 65]. However, deconvolution of the rate constants for the underlying microscopic steps using conventional global analysis was not possible without additional experiments under more controlled conditions (e.g., seeded aggregation) [66].

The variant‐specific coefficients correspond to any additional effects on aggregation beyond those accounted for by the general terms and reflect the structural context of the mutations (Figure 2d). We found that the effects of mutations in residues found in the disordered regions of αSyn fibrils (i.e., fuzzy coat highlighted in light gray in Figure 2a, KQ1 and KQ2) are well captured by the general term scaling (non‐significant values of β_KQ1_ and β_KQ2_). In contrast, the effects of mutations in residues resolved in fibril cores (highlighted in dark gray in Figure 2a) are significantly lower than expected from general term scaling, an effect which is most pronounced for K80 (KQ6) and K43+K45 (KQ4). We speculate that the distinct effects of these two classes of mutations reflect differences in the interaction networks of the affected residues. Residues in the fuzzy coat engage in fewer and more transient, electrostatically dominated contacts, whereas in the fibril core the loss of favourable electrostatic interactions may be partly compensated by the formation of new contacts mediated by glutamine residues.

Combining general, sequence‐specific, and experimental solution condition‐related effects allows us to compare and generalize combined mutational effects into three categories (Figure 2e). First, mutations in the N‐terminus (KQ1 and KQ2) significantly slow down aggregation relative to WT, arguably due to increased electrostatic repulsion between monomers as well as between monomers and fibrils. Second, the aforementioned variants KQ4 and KQ6 aggregate faster, or with comparable kinetics as WT under most solution conditions studied, indicating an altered interaction network of residues 43, 45 and 80 upon their mutation. Finally, variants KQ3, KQ5, and KQ7 exhibit aggregation kinetics that are comparable to or slower than WT, with their behaviour dependent on the solution conditions.

### 2.3 Monomer Compaction is a Poor Predictor of Half‐Time Scaling

The regression model described above captured both the general and sequence‐dependent effects reasonably well. Consistently, we find that the effects of the charge modulations persist even at 300 mM salt, where long‐range electrostatic interactions are screened (Debye length < 1 nm), suggesting that modified local interactions beyond global electrostatic repulsion can be responsible for the altered aggregation propensity. To investigate the effect of mutations on the conformational space of αSyn monomers, we complemented our experimental analysis with coarse‐grained molecular dynamics simulations of WT and KQ cluster variants using the CALVADOS2 force field (Figure S4) [67]. Across individual variants, we observed a negative correlation between aggregation half‐times and single‐chain compactness (radius of gyration R_g_, Figure S4d). However, neither R_g_ nor other ensemble‐averaged descriptors captured the global variation in experimental aggregation kinetics of all variants (R^2^ = 0.1, Figure S4d). In particular, the half‐time scaling at physiological salt concentration remained unexplained by monomer ensemble size, as all variants sampled predominantly extended conformations yet exhibited widely differing aggregation kinetics (half‐times of ∼3‐260 h, Figure 2; Figure S4c, d). Together, these findings suggest that while single‐chain compaction is somewhat correlated with overall aggregation kinetics, the sequence dependence of the KQ mutational effects arises from altered localized intermolecular interactions rather than monomer conformational properties. This finding reflects the fact that the free energy landscape of an isolated monomer is distinct from that of a monomer in contact with another monomer or a fibril end, due to the additional interactions via intermolecular contacts.

### 2.4 Variants With KQ Mutations Near the Fibril Core Form Stable Fibrils that are not Efficiently Elongated by Wild Type αSyn

While aggregation kinetics primarily report on the transition states of the aggregation reaction, equilibrium thermodynamics provides a broader view of the energetics of the resulting fibrils. We therefore investigated the effects of KQ mutations on αSyn fibril polymorphism through analyses of fibril thermodynamic stability and seeding properties. We prepared fibrils from all KQ cluster variants under the conditions at which WT structures of polymorphs 2a and 2b have been solved previously using cryo‐EM microscopy (Table 4) [68].

**TABLE 4:** Conditions used for assembly of different fibril polymorphs used in this work.

Fibrils	Buffer	T (°C)	Shaking (rpm)	Time (days)
WT—Fm	50 mM Tris‐HCl 150 mM KCl pH 7.4	37	600	7‐84
WT—Ri	5 mM Tris‐HCl pH 7.4	37	600	14
KQ	50 mM Tris‐HCl 150 mM KCl pH 7.4	37	600	14‐28

**FIGURE 3:**
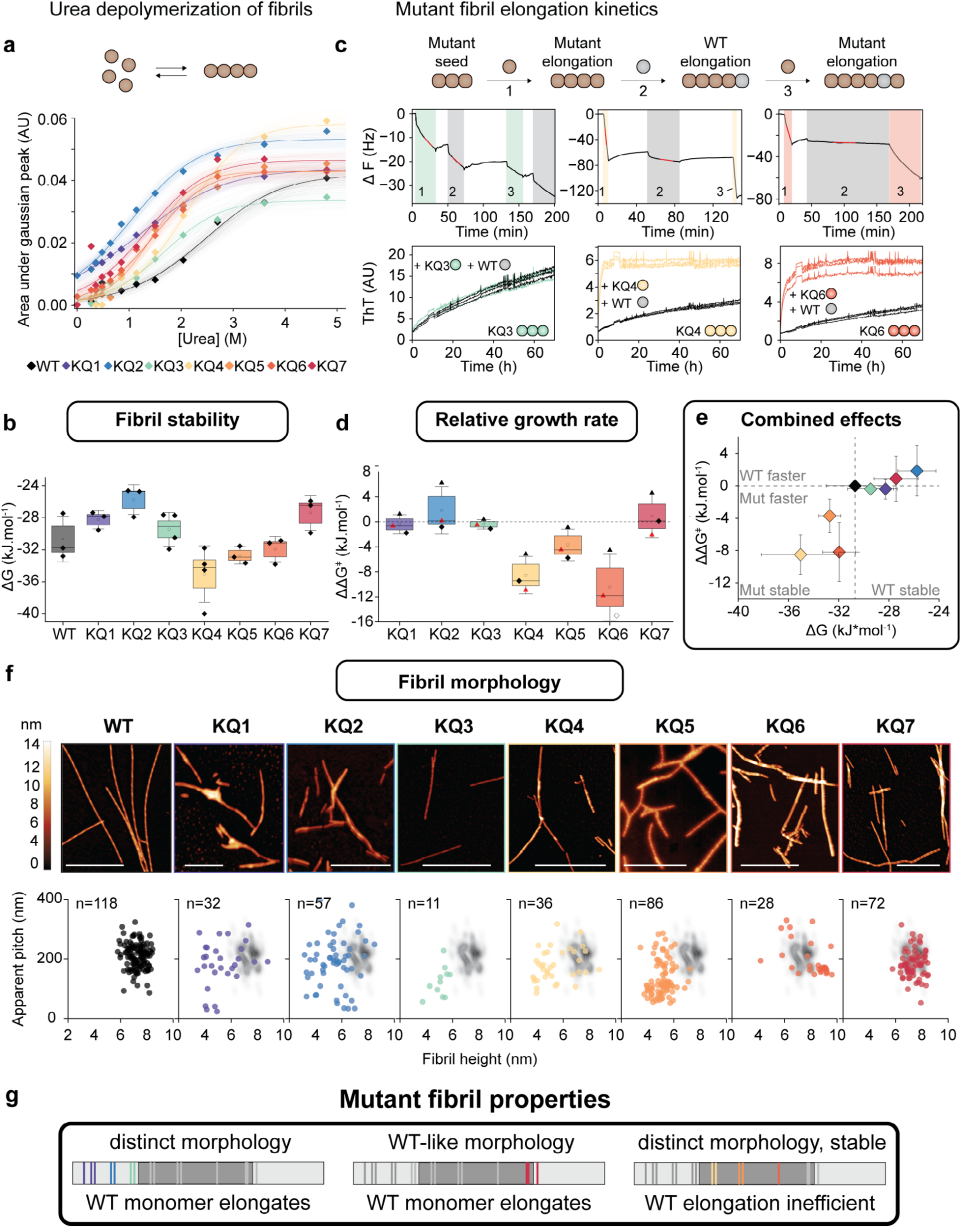
Analysis of fibrils formed by KQ cluster variants. a) Urea depolymerization of fibrils formed by KQ cluster variants. The monomer concentration in equilibrium with fibrils at increasing concentrations of urea was determined from the area under the Gaussian peak (y‐axis) using transient incomplete separation in laminar flow [69]. The data were fitted to the isodesmic polymerization model using Markov‐chain sampling of solutions as described in our previous study [71]. Hundred randomly selected solutions from a total of 2000 sampled solutions are shown as curves with the best solution highlighted in bold. b) Stability of WT and KQ fibrils obtained from fitting the urea depolymerization experiments. The mean and median values from three independent measurements are depicted by open squares and line, respectively. The SE and SD (n = 3) are visualized as box and whiskers, respectively (Table S3). The individual depolymerization curves and correlation plots between ΔG and the m‐values are shown in Figure S5. (c) Elongation kinetics of KQ variant fibrils monitored by ThT fluorescence and quartz crystal microbalance (QCM). (Top and middle) QCM experiments of KQ fibril growth. Immobilized variant fibrils were first allowed to elongate by variant monomers (colored boxes), followed by a washing step and elongation by WT monomers (grey boxes). The elongation rates of the variants and WT were quantified from the slopes (highlighted in red) of the changes of the third harmonic overtone frequency (black lines) during the first and second injections, respectively. (Bottom) Comparison of aggregation kinetics of 80 µM WT (black) and KQ3 (green), KQ4 (yellow), or KQ6 (red) monomers in the presence of 2.5 µM respective variant seeds monitored by the ThT fluorescence assay. Aggregation of all variants together with analysis of monomer conversions to fibrils are shown in Figure S6. (d) Relative growth rate (ΔΔG^ǂ^) of mutant variants and WT monomers on fibrils of KQ cluster variants. Negative ΔΔG^ǂ^ values indicate that KQ seeds are elongated faster by their respective monomers compared to the WT monomer. The black and red symbols represent values derived from ThT and QCM experiments, respectively. The mean, median, SE, and SD of three independent measurements are depicted by open squares, line, box and whiskers, respectively. The open symbol in KQ6 corresponds to a dataset where elongation of WT was not observed. e) Comparison of stability (ΔG) and relative growth rate (ΔΔG^ǂ^) of KQ fibrils. Datapoints and error bars correspond to the mean ± SE from b and d. f) AFM analysis of elongated fibrils. The apparent pitch lengths (i.e., dominant frequency along the main fibril axis) and heights were extracted from the manually selected fibril profiles using an automated python script [54, 71]. Kernel density estimation was applied to the morphological fingerprint (i.e., height vs pitch plot) of WT seeds elongated by WT monomer for better visualization. Results from fibril height analysis together with all values related to this figure are provided in Table S3.

First, we used urea depolymerization and microfluidic transient incomplete separation to measure the thermodynamic stability of the fibrils (Figure 3a,b; Figure S5) [69]. We used Bayesian inference with Markov Chain Monte Carlo (MCMC) sampling to fit the data and to verify that no parameter correlation between Gibbs free energies (ΔG) and denaturant m‐values obscured our conclusions (Figure S5) [70, 71]. Fibrils formed by variants carrying mutations outside or at the periphery of the resolved fibril cores (KQ1, KQ2, KQ3 and KQ7) [72] exhibited stability comparable to or lower than that of the WT. In contrast, variants with mutations of residues resolved in αSyn fibril core structures (KQ4, KQ5, and KQ6) formed fibrils with higher stability than the WT under the tested solution conditions (Figure 3b; Table S3).

Next, we investigated how changes in fibril stability are related to their respective growth rates. We quantified the changes to the free energy barriers of elongation (ΔΔG^ǂ^) from relative growth rates of mutant and WT monomers on the fibrils formed by KQ cluster variants using both seeded ThT and QCM biosensor experiments (Equation (M3), Figure 3c,d) [54]. This strategy avoids complications that could stem from variations in the number of growing fibril ends between experiments (see method section for details). We observed little difference between elongation of KQ1, 2, 3, and 7 fibrils by WT and the respective mutant monomers. In contrast, variants KQ4, KQ5 and KQ6, which carry mutations of residues resolved in the known fibril cores, showed distinct behavior. Specifically, WT monomer was inefficient in elongating their seeds, especially those formed by KQ4 and KQ6, even though elongation by their respective KQ monomers was fast.

**FIGURE 4:**
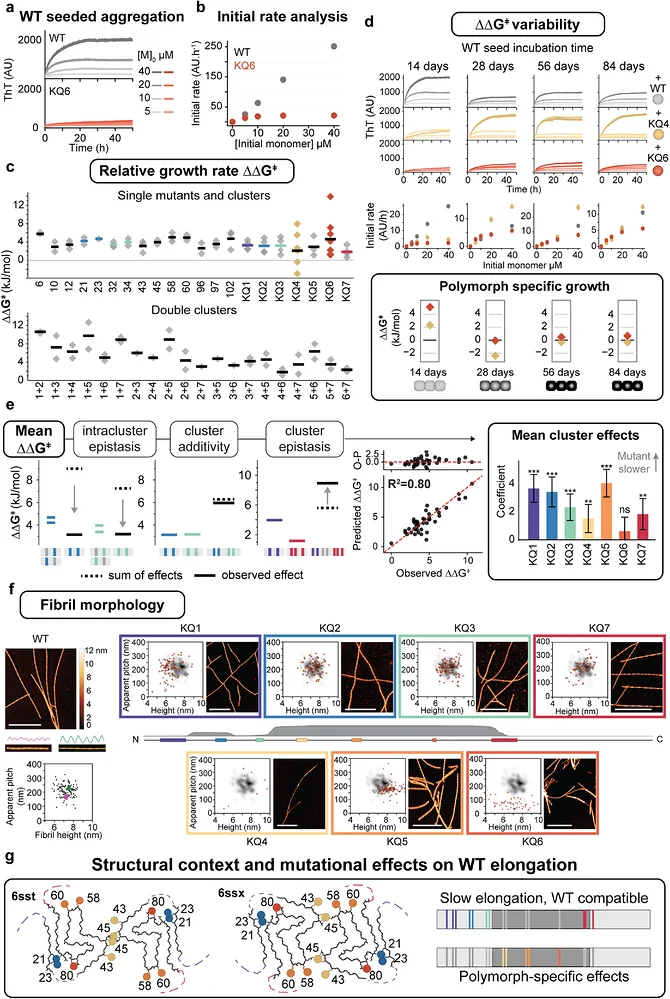
Effects of KQ mutations on αSyn WT fibril elongation. (a) Example of seeded aggregation kinetics of WT (top) and KQ6 (bottom) monomers in the presence of 2.5 µM WT‐Fm seeds monitored by ThT kinetics. The raw data for all variants are shown in Figures S9–S13. (b) Initial rate analysis. The initial rates were obtained from linear fitting of the raw ThT data (a) in the 0 – 2.5 h range. The determined initial slope of each curve was used to derive the relative growth rate and change in free energy barrier (Equation M3). (c) Relative free energy barriers for fibril elongation (ΔΔG^ǂ^) derived from the ratio of apparent growth rates between WT and mutant monomers. Positive ΔΔG^ǂ^ values indicate that elongation by WT is faster than by the mutant monomers. The averaged values from independent measurements (diamonds) are shown as lines. (d) Investigation of variability of ΔΔG^ǂ^ for selected mutants KQ4 (K43Q + K45Q) and KQ6 (K80Q) using different batches of WT seeds [71]. (Top) ThT experiments with 2.5 µM WT‐Fm seeds in the presence of 5, 10, 20, and 40 µM monomers (indicated by respective colour gradients). (Middle) Initial (0‐2.5 h) slopes from the ThT kinetics as a function of initial monomer concentration. (Bottom) ΔΔG^ǂ^ of KQ4 (yellow) and KQ6 (red) as a distinguishing feature of WT seeds of varying maturation age. Values here were derived from linear fits of the initial rate plots in the 0–20 µM monomer range. (e) Weighted least square regression of mean ΔΔG^ǂ^ values shown in (c). (Left) The non‐additivity of effects of individual mutations within one cluster (left scheme) is demonstrated for KQ2 (= K21Q+K23Q, blue) and KQ3 (= K32Q+K34Q, green), additivity between clusters (middle scheme) is shown for variant 2+3 (= KQ2+KQ3), and example epistatic effect between clusters KQ1 (purple) and KQ7 (red) is depicted for variant 1+7 (right scheme). (Middle) Correlation between measured and predicted ΔΔG^ǂ^ values from the additive model with termini coupling (Equation 7). The adjusted R^2^ value is shown together with the line with a slope of 1 (red) and residuals (observed‐predicted, top). (Right) Cluster‐specific coefficients obtained from the model. Error bars indicate ± 95% confidence intervals. Asterisks denote levels of statistical significance: *p < 0.05; **p < 0.01; ***p < 0.001, ns – not significant. (f) AFM analysis of products from elongation of WT‐Fm seeds. The apparent pitch lengths (i.e., dominant frequency along the main fibril axis) and heights were extracted from the manually selected fibril profiles using an automated python script (example profiles shown as pink and green lines and corresponding dots for WT) [54, 71]. Kernel density estimation was applied to the morphological fingerprint (i.e., height vs pitch plot) of WT seeds elongated by WT monomer for better visualization. Each red dot corresponds to the profile of a single fibril. White scale bars correspond to 1 µm. The sequence profile in the middle (grey) corresponds to the frequency of αSyn residues resolved in fibril cores. (g) Structural context of the mutational effects on WT fibril elongation. (left) Cross‐sectional area of fibril polymorphs 2a (6ssx) and 2b (6sst) resolved previously under similar solution conditions, highlighting the lysine residues [68]. (right) Classification of KQ cluster variants according to their effects on WT seed elongation highlighting the structural context of the residues (disordered regions – light grey, fibril cores – dark grey).

To resolve these differences, we determined the height and apparent pitch length of the elongated fibrils using atomic force microscopy (AFM) (Figure 3f; Figure S7). The WT fibrils displayed two distinct populations characterized by shorter (ca. 160 nm, pink box in Figure 4f) and longer (ca. 220 nm, green in Figure 4f) apparent pitch lengths with heights of 6–8 nm. The KQ7 variant formed WT‐like fibrils, whereas KQ5 fibrils had shorter helical pitch (∼100 nm). The rest of the variant fibrils appeared mostly flat or exhibited irregular patterns along their main axis (apparent pitch < 100 nm). The lack of twist in KQ4 and KQ6 fibrils was further confirmed by TEM, which revealed a dominant population of flat fibrils (Figure S8). Fibrils formed by KQ1, KQ2, KQ3, and KQ5 variants exhibited lower heights (3–6 nm) compared to WT fibrils, suggesting either their tighter packing or that they are formed by a single protofilament (Table S3).

Altogether, we observed that the effects of the K‐to‐Q mutations on fibril stability are governed more strongly by their position within the sequence than their total number. The flat morphology of most of the resulting fibrils indicates that the fibril twists are modulated by interactions between N‐ and C‐termini within the fibrils [73]. Our findings that lysines in proximity to the fibril core (K43, K45, K58, K60, K80 and K96) modulate the fibril morphology and stability is consistent with high‐resolution structural characterization of αSyn WT fibrils, where these residues are found to stabilize protofilament interfaces by forming salt bridges or binding polyanionic molecules [72, 73, 74, 75, 76, 77, 78, 79, 80, 81]. The link between variant fibril stability and their ability to be efficiently elongated by WT monomer (Figure 3e) are compelling biophysical signatures of a fibril polymorphism change. Although the absence of a well‐defined helical pitch precludes structural resolution of the underlying interaction networks stabilizing the fibrils, their incompatibility with WT monomer elongation likely arises from the energetic penalty associated with incorporating lysine residues into fibril cores where glutamines are present. Recently solved structures of fibrils formed by acetylated K80 revealed that this posttranslational modification disrupts salt bridges found in WT polymorphs either with E35 (Lewy body fold, 8a9l), E46 (MSA fold, 6xyo) or E83 (Polymorph 2b, 6rtb) and instead leads to packing of ^ac^K80 to a hydrophobic pocket formed by A69 and V71 (9ptc) [43]. This provides possible structural context supporting our conclusions regarding incompatibility of WT monomers with K80Q and possibly other mutant fibrils (KQ4, KQ5). Whilst our AFM analysis indicates a shift in polymorph distribution based on fibril ultrastructural morphology, it is mostly insensitive to modest changes in polymorph folds that do not significantly alter the protofilament packing [43]. Fibril stability and relative growth‐rate measurements may therefore provide more broadly applicable, albeit indirect, biophysical inference of fibril polymorphism on multiple structural levels.

### 2.5 Lysine Mutations in Monomeric αSyn Impair WT Fibril Growth in Residue‐Specific Manner

We established that the effect of KQ mutations on the αSyn free energy landscape is sequence specific, yet it remained unclear whether the differential seeding competence is governed more by the intrinsic structural properties of the fibrils, or the monomers. To investigate this, we prepared seeds from WT fibrils formed in solution conditions characterized previously (WT‐Fm, [68, 69, 71]) and analyzed the elongation kinetics of 41 variants carrying one to six mutations using a ThT assay (Figure 4a,b; Figures S9–S13) to assess how the changes to the sequence of monomeric αSyn influence the relative fibril growth (ΔΔG^ǂ^, Figure 4c; Table S4).

Two clear patterns emerged from the relative growth rate analysis. First, elongation of the WT‐Fm seeds by mutant monomers was slower compared to elongation with the WT monomers (Figure 4c), except for a few repeats with KQ4. Second, the relative growth rates of certain variants (most noticeably for KQ4 and KQ6) appeared to be highly dependent on the batch of the WT‐Fm seeds. We investigated this unusual variance more systematically using WT‐Fm seeds of different maturation ages (2 weeks – 3 months) which we characterized previously to be morphologically heterogeneous (Figure 4d, [71]). We observed that the ΔΔG^ǂ^ of KQ4 and KQ6 were distinct for the 14 and 28‐day aged fibrils, and similar for the matured ones (2 and 3‐months old). Under the highly seeded assay conditions, elongation is the dominant process, governed by interactions that mediate the association and conversion of mutant monomers at WT fibril ends. Although we cannot fully exclude residue‐specific effects on monomer conformational ensembles in contact with fibrils, our results suggest that the relative growth rates are mainly influenced by the polymorph composition of the seeds. We therefore hypothesize that the observed pronounced variance in their ΔΔG^‡^ values arises from the underlying polymorph heterogeneity combined with variant‐specific polymorph selectivity. This indicates that K43, K45 and K80 are critical for recognizing specific αSyn fibril folds in line with other reports [43, 49].

We further investigated the dependence of the average ΔΔG^‡^ values on the relevant experimental parameters (Figure 4e; Table S4). In contrast to the unseeded aggregation, we found that the monomer net charge (i.e., number of mutations) explained only ∼35% of the observed variance (Equation 5); figure S14a), meaning that sequence and/or structural context of the mutations in monomers plays an important role for the ability to elongate WT fibrils. We therefore assumed that mutational effects can be decomposed into a global contribution associated with the overall mutational burden, cluster‐specific contributions, and potential interactions between clusters. To model this residue dependency, we used cluster‐specific coefficients representing the average contribution from each KQ cluster (i.e., either single mutation or whole cluster mutated, Equation (6) with ∑β_c_ = 0, adjusted R^2^ = 0.69, Figure S14b).

(5) $$\Delta \Delta G^{‡}=\beta_{0}+\beta_{m}\#muts+\varepsilon$$

(6) $$\Delta \Delta G^{‡}=\beta_{0}+\sum_{j=1}^{7}\beta_{j}KQ_{j}+\sum_{j=1}^{7}\beta_{c}coupling+\varepsilon$$

(7) $$\begin{array}{cc} \sum \beta_{c}coupling & =\beta_{12}KQ1.KQ2+\beta_{17}KQ1.KQ7\\ & \;+\beta_{27}KQ2.KQ7+\beta_{57}KQ5.KQ7 \end{array}$$

where ε is the residual error, β_0_ is the baseline intercept centered around zero (corresponding to WT), #muts is the number of mutations with coefficient β_m_, KQ_j_ and couplings are binary encodings for each cluster or their combination with respective coefficients β_j_ and β_c_.

This model captured well the two main trends observed for ΔΔG^‡^ values across the variants. First, the effects of single‐point mutations within individual clusters were non‐additive (Figure 4e). Second, the combined effect of mutating two distinct clusters was in most cases well approximated by the sum of their individual effects (Figure 4e). The contrast between non‐additive and additive effects of intra‐ and inter‐cluster mutations, respectively, can be explained through compensatory effects of nearby mutations. This is reminiscent of the modular organization observed in protein–protein interactions [82], suggesting analogous principles underlying monomer–fibril recognition. Additionally, we observed that the terminal clusters (KQ1, KQ2, KQ7) show significant epistatic effects indicating that interaction of the termini is important for monomer‐fibril interactions and fibril growth (Eq. 6+7, adjusted R^2^ = 0.8, Figure 4e).

The magnitude of the model coefficients for clusters KQ1, KQ2, KQ3, and KQ5 underscored their marked effect on reducing WT seed elongation efficiency, whereas the other clusters had weaker or statistically insignificant contributions (Figure 4e). The strong effects of K‐to‐Q mutations outside of the fibril core (i.e., KQ1, KQ2, K6Q, K102Q, Figure 4c) highlight the important role of the electrostatic interactions of the fuzzy coat in the elongation kinetics of αSyn fibrils [51, 83, 84]. In comparison, the large variation of ΔΔG^ǂ^ values observed for variants with mutations near core (KQ4 and KQ6, Figure 4d) reflects their sensitivity to the specific folds of fibril polymorphs. Reduced efficiency was previously observed for elongation of K80A mutant monomer on seeds from N‐terminally acetylated αSyn fibrils [49], as well as for WT seeded aggregation of acetylated K12, K43, and K80 monomers [43].

Our conclusions are consistent with the AFM analysis of the fibrils elongated by different monomers (Figure 4f). We observed WT‐like morphological fingerprints for seeds elongated by monomer variants with mutations in the fuzzy coat (KQ1, KQ2, KQ3, and KQ7), whereas elongation by variants with mutations in the core resulted in only subpopulations of fibrils with short apparent pitch (KQ5) or mostly flat fibrils (KQ4, KQ6) (Figure 4f; Figure S7). Dominant population of mostly flat fibrils or fibrils with slight twist were also found during structural analysis of WT seeded fibril growth by acetylated K43 and K80 monomers, in line with our findings here with acetyllysine‐mimicking K‐to‐Q mutations [43].

Together, our results show that the sequence features of the monomer play a crucial role in templated aggregation and highlight critical roles of lysines in shaping the complex polymorphic landscape of αSyn. Specifically, lysines in the disordered regions (K6, 10, 12, 21, 23, 32, 34, 96, 97, 102) primarily facilitate electrostatically driven monomer‐fibril interactions, whilst the others (K43, 45, 58, 60, 80) form local electrostatic networks that play roles in shaping the folds of the fibril cores (Figure 4g).

### 2.6 Residue‐Specific Effects of Several KQ Mutations Enable Polymorph Discrimination in Low‐pH Seed Amplification Assay

**FIGURE 5:**
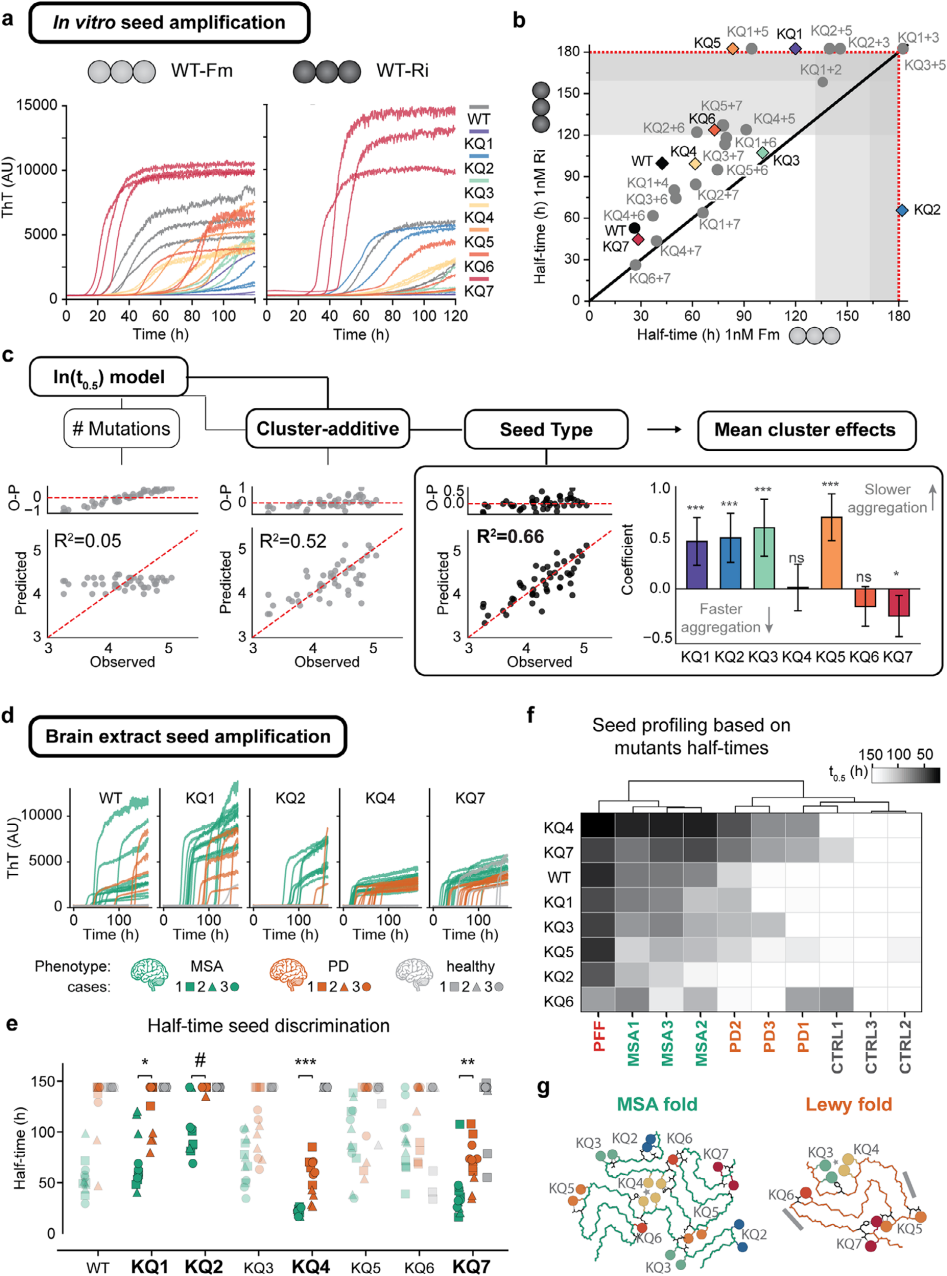
Effects of KQ mutations on seed amplification of WT polymorphs at low pH. (a) ThT kinetics of WT and KQ cluster variants in the presence of 1 nM WT‐Fm (left) or WT‐Ri (middle) seeds. The seeded aggregation kinetics of all variants carried out in the presence of 0, 1 nM, and 1 µM of sonicated WT seeds are shown in Figures S15 and S16. (b) Correlation between aggregation half‐times in the presence of 1 nM Fm and Ri seeds. The values correspond to the means of the half‐time values obtained from fitting the raw data from experiments carried out in triplicates in two independent measurements (circles, diamonds). The grey zones correspond to the endpoints of the measurements and points within these zones were derived with lower confidence. The points outside of the red lines correspond to the variants whose ThT signal did not plateau during the experiments and are shown for illustration. The black line has a slope of 1 to easily visualize the specificity of the mutant monomers to different WT polymorphs. (c) Weighted least square regression of log transformed half‐times using Equation (8) (# mutations), Equation (9) with β_f_ = 0 (cluster‐additive), and full Equation (9) (Seed type). Individual points correspond to the mean values from panel (b) with a line with slope of 1 depicted in red. Residuals (observed‐predicted) are shown on top. (Right) Variant‐specific coefficients β_j_ from Equation (9) representing the average contribution when a whole cluster is mutated. Values correspond to the mean ± 95% confidence intervals. Asterisks denote levels of statistical significance: *p < 0.05; **p < 0.01; ***p < 0.001. (d) Seed amplification of brain homogenates from patients with Parkinson's disease (PD, orange), multiple system atrophy (MSA, green), and healthy controls (grey) using selected monomer substrates. (e) Half‐time analysis of the SAA. The half‐times were derived from raw data shown in (d) and Figure S17 using Equation (M1) with an extra parameter accounting for a sloping top baseline. Each sample (n = 3, squares, circles and triangles) was run in quadruplicates (Table S6). Statistical analysis was evaluated using a Mann‐Whitney U‐test for comparison between MSA and PD. All negative datapoints (annotated as 144 h, i.e., endpoint) were excluded from statistical analysis. Asterisks denote levels of statistical significance: *p < 0.05; **p < 0.01; ***p < 0.001, # – too few datapoints for analysis. (f) Hierarchical clustering of seeds based on mutational effects on half‐times. Each value represents the average half‐time from quadruplicate measurements for the indicated mutant and seed. Missing values were assigned the maximum half‐time value of 144 h before averaging. Mutants and seeds were clustered using Euclidean distance and Ward linkage. Closer clustering indicates more similar mutational response profiles across seeds. (g) Cross‐section of the representative MSA (6xyo) and PD (8a9l) fibril folds. The position of lysines mutated in each KQ cluster variant is highlighted by circles.

Motivated by the distinct mutational effects on elongation, we investigated whether similar differentiation can be observed for seed amplification under conditions where secondary nucleation plays a dominant role, i.e. at acidic pH (Figure 5) [58]. We used the solution conditions established in our recently developed seed amplification assay (SAA) in which aggregation is dominated by secondary nucleation (i.e., quiescent, pH 3, 250 mM Na_2_SO_4_, Table 5) [85] and followed aggregation of WT or mutant monomers in the presence of two distinct types of WT seeds, WT‐Fm and low‐salt polymorph WT‐Ri, at 1 nM concentration in monomer equivalents (Figure 5a; Figures S15 and S16).

**TABLE 5:** Overview of the conditions for different ThT assays used in this study. Conc. – assay with varying initial monomer concentration, S.O. single‐orbital, D.O. – double orbital, NB – non‐binding, NT – non‐treated, SAA – seed amplification assay.

Assay	Buffer	[Protein] µM	Bead	Shaking (rpm)	Corning plate	Data Figure
*De novo* – large scale						
Conc.	50 mM Tris‐HCl 150 mM KCl pH 7.4	variable	3 mm Si_3_N_4_	S.O. (600)	NB‐96w 3651	2a, SI‐2,19
pH	50 mM Na‐citrate, pH 4.6, 5.4, 6.2, and 7.4	50	3 mm Si_3_N_4_	S.O. (600)	NB‐96w 3651	2a, SI‐2,19
Salt	10 mM NaPi 0, 50, 150, 300 mM KCl pH 7.4	50	3 mm Si_3_N_4_	S.O. (600)	NB‐96w 3651	2a, SI‐2,19
*De novo* – small scale						
Conc.	50 mM Tris‐HCl 150 mM KCl pH 7.4	Variable	1 mm glass	D.O. (300)	NT‐384w 3540	SI: 18a
pH	50 mM Na‐citrate, pH 4.6, 5.4, 6.2, and 7.4	50	1 mm glass	D.O. (300)	NT‐384w 3540	SI: 18b
Salt	10 mM NaPi varying salts pH 7.4	40	1 mm glass	D.O. (300)	NT‐384w 3540	SI: 18c
Seeded kinetics						
Elongation WT‐Fm KQ	50 mM Tris‐HCl 150 mM KCl pH 7.4	5‐80	—	—	NB‐384w 3544	4, SI: 6,9‐12
SAA	50 mM Na‐citrate 250 mM Na_2_SO_4_ pH 3	10	—	—	NB‐384w 3544	5, SI: 15–17

The extracted half‐times from the aggregation kinetics of 26 αSyn variants were positively correlated between the two types of WT seeds, with a few noticeable outliers (Figure 5b). We used linear regression of the log‐transformed aggregation half‐times to deconvolute the features underlying the aggregation behavior (Figure 5c; Table S5). Similarly to relative fibril growth at neutral pH, the mutational effects were residue‐specific (adjusted R^2^ = 0.05 for the model with only global effects, Equation (8)), and the variance could be captured relatively well by the additive model with cluster‐specific coefficients (Equation 9 with β_f_ = 0, adjusted R^2^ = 0.52). In contrast to the relative growth rate at neutral pH, we did not find any significant couplings between clusters, however, accounting for the fibril type significantly improved the quality of the model (adjusted R^2^ = 0.66). The positive coefficient for Ri fibrils (β_Ri_ = 0.4) compared to the Fm baseline reflects the overall slower kinetics observed for weakly seeded experiments with this polymorph under acidic conditions. Although this difference may arise from distinct surface properties of the two fibril types, we cannot exclude the possibility of a systematic deviation due to concentration differences introduced during fibril preparation and handling (estimated at 4–10%).

(8) $$\ln\left(t_{h}\right)=\beta_{0}+\beta_{m}\#\mathrm{muts}+\varepsilon$$

(9) $$\ln\left(t_{h}\right)=\beta_{0}+\beta_{f}\mathrm{Fibr}\mathrm{il}+\sum_{j=1}^{7}\beta_{j}KQ_{j}+\varepsilon$$

where *Fibril* is the binary encoding for fibril type (i.e., WT‐Fm or WT‐Ri).

Interestingly, the pattern of cluster‐specific contributions was similar compared to the relative growth rates at neutral pH even though the fitted coefficients differed in magnitude and sign between the two assays (Figure 4e and Figure 5c). N‐terminal (KQ1–KQ3) and KQ5 clusters impaired the aggregation, whereas KQ4 and KQ6 had effects similar to WT and KQ7 exhibited moderately accelerated aggregation (Figure 5c). This result suggests that secondary nucleation under low‐pH and elongation at neutral‐pH are driven by interactions between similar sequence regions. However, there is still considerable unexplained variance in the data which means that some mechanistic aspects are not captured by the model. We hypothesize that this arises from the convoluted nature of the half‐time parameter, which is primarily governed by the rates of secondary nucleation and elongation.

Notably, amplification of the seeds by a handful of variants was specific to the polymorph type. Specifically, variants KQ1 and KQ5 amplified Fm, but not Ri fibrils, whereas the opposite trend was observed for the variant KQ2 (Figure 5b). Interestingly, KQ2 is a double‐point mutant that includes K23Q, a mutation commonly used as a substrate in the state‐of‐the‐art SAA protocols [86, 87].

To investigate whether the polymorph specificity of some of our designed variants can be used in a similar manner practically in SAAs, we used the KQ cluster variants to amplify seeds from brain homogenates of patients with Parkinson's disease (PD, n = 3) and multiple systems atrophy (MSA, n = 3), as well as healthy controls (n = 3) (Figure 5d; Figure S17, Table S6). We minimized the effects of primary nucleation prior to the start of the experiments by filtering the monomers through 100‐kDa spin‐columns, using tips with filters, and overlaying each sample with fluorinated oil with PEG‐based surfactant, which helped achieve aggregation‐free negative controls (where PBS buffer was added instead of brain homogenate) [85]. The addition of minute amounts of diseased brain‐derived samples (10^5^‐fold buffer dilution) triggered aggregation significantly more compared to those from healthy controls (Figure 5e). Overall, amplification proceeded more rapidly in MSA than in PD samples, reflecting the more aggressive nature of the underlying pathology. The half‐time scaling trends in brain‐derived samples closely mirrored those observed for the in vitro PFFs (pre‐formed fibrils), with KQ1, KQ2, KQ3, and KQ5 showing slower kinetics relative to the faster KQ4, KQ6, and KQ7 variants. Importantly, variants KQ1, KQ2, KQ4 and KQ7 showed significantly higher sensitivity toward MSA samples compared to those from PD brains (p < 0.05, Figure 5e), highlighting their potential use in SAA for distinguishing between MSA‐ and PD‐associated polymorphs. However, further testing, including seed dilution series and increased number of biological samples is needed to confirm selectivity and exclude effects from variable seed load between the samples.

Rationalization of our observations within the structural context of the lysines in the known MSA (pdb id: 6xyo) and Lewy body (PD) (pdb id: 8a9l) fibril folds is challenging (Figure 5g). Residues mutated in three out of the four variants that were most discriminatory in our SAA assays (K6, 10, 12 – KQ1, K21, 23 – KQ2, and K96, 97, 102 – KQ7) are nearby or within the disordered regions of the fibrils. KQ1 and KQ2 variants show opposing trends for in vitro PFFs compared to similar trends in brain‐derived samples, suggesting distinct organization of the fuzzy coat in each fibril type. Residues K43 and K45 are in contact with unknown electron densities in the structures of both disease polymorphs. Efficient amplification of such folds with KQ4 variant in which both lysines are mutated suggest that the introduced glutamines can stack in the ladder stabilizing the overall structure even in the absence of the unknown cofactor. However, given the conditions of our SAA assay, robust structural analysis of the amplified products is needed to provide additional mechanistic understanding of why the different variants elongate disease‐derived fibrils differentially. Such analysis can provide bases for rational design of mutations that can selectively and faithfully propagate disease‐relevant fibril folds.

Nevertheless, our findings suggest that the effects of the mutations are governed by complex polymorph‐specific features that can be exploited for fibril discrimination. The use of multiple mutants in a combined SAA to distinguish disease‐specific polymorphs has the potential to be more robust than relying on a single monomer substrate, as the relative trends are largely insensitive to the amount of seed‐competent material present in the samples. To demonstrate this, we carried out seed profiling based on the different variants’ half‐times (Figure 5f). Clustering broadly recapitulated the biological origin of the samples and suggested diagnosis‐associated grouping. However, this separation was driven largely by the generally slower and less frequently completed reactions observed for PD samples relative to MSA samples. The limited number of completed reactions, particularly for PD samples, precluded robust statistical evaluation of mutant selectivity. Nevertheless, several variants showed qualitatively promising results based on their per sample differences in half‐times relative to WT. Most notably, KQ5 aggregated faster than WT in two of the three PD samples, whereas it aggregated consistently more slowly than WT in all three MSA samples. Although this observation did not constitute statistically significant evidence of selectivity, it highlights the potential of a “mutant fingerprinting” approach in which relative, rather than the absolute half‐times, are used to characterize and potentially distinguish disease‐associated seeds. Our preliminary results demonstrate the principal feasibility of such an approach which we aim to explore in greater detail in the future.

## 3 Discussion

### 3.1 Regression Analysis as a Framework for Isolating Mutational Effects Across Experimental Conditions

In this study, we carried out a comprehensive mutational analysis of αSyn to (i) investigate sequence specific effects of acetyllysine‐mimicking mutations, (ii) quantify how electrostatic interactions contribute to the kinetics and thermodynamics of its assembly, and (iii) assess the broader applicability of this sequence‐perturbative approach for probing the degenerate energy landscapes characteristic of self‐associating IDPs.

Several systematic mutational studies aiming to elucidate sequence determinants of αSyn aggregation have been carried out (see, for example [88], for their review). However, their global mechanistic interpretation is often difficult, due to the reported aggregation half‐times or rates stemming from experiments that have been conducted under conditions influenced by many variables—such as shaking speed, the presence of beads, buffer composition, reaction vessel size, and, importantly, the existence of multiple aggregation pathways. These factors can significantly impact the observed aggregation behavior, making it difficult to isolate sequence‐specific effects and carry out quantitative comparison between results from different studies.

Here, we propose global regression analysis of aggregation as a framework that offers several key advantages over direct comparison of absolute aggregation half‐times. First, it enables disentangling of the intrinsic parameters affecting αSyn aggregation from the influence of experimental conditions. To demonstrate this idea, we compared the experimental results measured using a “conventional protocol” for measuring aggregation kinetics (i.e., 96‐well plate 100 µL/well, single 3 mm bead, continuous shaking, Figure 2) to those measured at smaller scale (384‐well plates, 15 µL/well, 1 mm bead, 30s shaking in 10 min intervals, Table 5, Figure S18) with independent protein batches. While the comparison of absolute half‐times is not accurate (Figure S19d), the model coefficients from the two experimental datasets correlate strongly (Figure S19e, f and Table S7), supporting similar interpretation of both datasets. The framework is therefore, in principle, well suited for comparing results from aggregation experiments across different laboratories and experimental protocols. The model can readily be extended to incorporate additional parameters, such as aromaticity, hydrophobicity, or secondary structure propensity, provided that sufficient data from mutants probing these physicochemical properties become available.

### 3.2 Deviation From Global Scaling can Help Identify Mutations With Distinct Mechanistic Behavior

Importantly, our framework enables quantitative testing of various mechanistic hypotheses. As an example, we investigated whether the general physical principles derived from the KQ dataset (Equation 2) can explain the aggregation behavior of charge‐altering familial mutations (E46K, H50Q, G51D, and A53E) measured under identical experimental conditions (pH, salt and concentration assays, conventional protocol, Figure S20). We found that the aggregation of H50Q and A53E is well described by the general scaling, showing minimal deviation from predicted effects of charge, ionic strength, and protein concentration (Figure S20g). In contrast, E46K and G51D exhibit pronounced deviations from the model, aggregating more slowly than predicted by the general scaling.

The deviations identified in our study in the unseeded aggregation assays (E46K, G51D, KQ4, KQ6, KQ7) can serve as priors for subsequent hypothesis testing to determine why certain mutations behave as mechanistic outliers. Specifically, whether their effects arise from structural context, the nature of the substituted residues, or a combination of both, potentially leading to altered dominant aggregation pathways. Indeed, fibril structures of familial mutants as well as acetylated K80 αSyn reveal that altered local interaction networks involving the mutated residues can give rise to distinct fibril folds or protofilament packing arrangements [74, 75, 77, 78]. Our analyses suggest that similar effects may occur in fibrils formed by the KQ4 and KQ6 variants (Figures 2, 3, 4), providing a possible mechanistic basis for their deviations from the general scaling behaviour. The same framework can be extended to other physiologically or pathologically relevant αSyn modifications, including PTMs and PTM mimics, to identify residue‐specific effects with potential mechanistic or therapeutic relevance.

### 3.3 Translation of KQ Mutational Effects Into Energy Perturbation of αSyn Amyloid Landscape

Having established the utility of the regression framework, we next examined how the observed mutational effects reshape the αSyn aggregation energy landscape. Overall, our analysis shows that electrostatic interactions driving αSyn aggregation from homogeneous monomer solutions can be modelled reasonably well (72% of variance) by the expected global scaling of the relevant intrinsic properties, such as net charge and protein concentration, and extrinsic properties, such as ionic strength. In contrast, residue‐specific mutational effects become significant under conditions where aggregation is governed by monomer–fibril interactions, and the energy landscape is dictated by the available fibril structure. We observe similar magnitudes of the energy changes (relative to the WT reference) for the fibril growth and fibril stability of the mutant variants (Figure 3e), most of which displayed distinct morphological features compared to WT fibrils. Unlike Φ‐value analysis which we used previously to gain insights into the transition state ensemble of SH3 fibril elongation [54], the structure of the KQ fibrils was not imposed by seeded growth using WT fibrils. Instead, we interpret the observed correspondence such that the loss of electrostatic interactions important for WT seed growth promotes the formation of new interaction networks, stabilizing distinct fibril polymorphs in some of the KQ variants.

In another set of experiments, where we did impose the structure of WT fibrils by using them as seeds, we were able to quantify the increase of the energy barrier of elongation for the KQ variants to be around 4–6 kJ/mol which is in a range comparable to disruption of a surface‐exposed salt bridge of folded proteins [89]. We find that changes on the order of 8–10 kJ/mol (corresponding to approx. two‐thirds of the overall height of the energy barrier [90] are sufficient to render variants essentially incapable of elongation of WT seeds and vice versa (Figures 3 and 4). The changes to the energy barrier are higher compared to stability perturbations predicted for single point mutations by FoldX on 47 representative WT polymorph structures (mean ΔG_0_ = 1.6 ± 0.9 kJ/mol) (Figure S21 and Table S8 [91]. Both experimental and in silico data exhibit substantial variability, consistent with the pronounced polymorphism of αSyn fibrils, which impairs a determination of whether contact changes arise during formation of the transition state or exclusively within the fibrillar state.

### 3.4 Clustering of Mutational Effects Highlights the Importance of Residue Structural Context Within Fibrils

**FIGURE 6:**
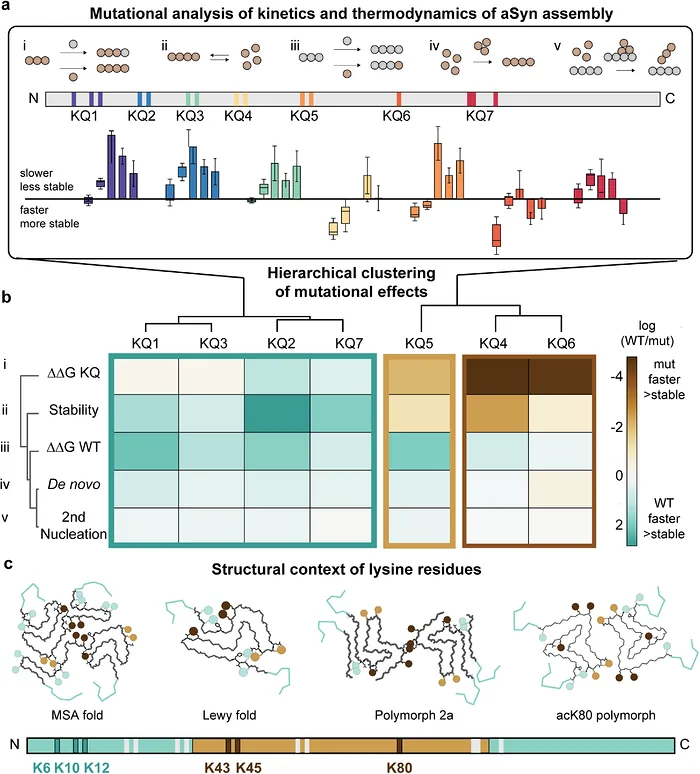
Hierarchical clustering of mutations based on the magnitudes of mutational effects. a) Schematic overview of the molecular aggregation pathways probed for the set of KQ cluster mutations and their effects. i) Relative growth rates from aggregation seeded by mutant fibrils (ΔΔG^ǂ^ KQ, Figure 3d), (ii) thermodynamic stability of KQ fibrils (Figure 3b), (iii) relative growth rates from aggregation seeded by WT‐Fm fibrils (ΔΔG^ǂ^ WT, Figure 4e), (iv) *de novo* aggregation (Figure 2), (v) seed amplification at low pH (secondary nucleation, Figure 5c). The bars correspond to mutational effects in these assays (from left to right). For *de novo* aggregation, half‐times were calculated from the model coefficients for the condition of 10 mM NaPi, 150 mM KCl, 50 µM αSyn pH 7.4. b) Hierarchical clustering of mutational effects. Relative effects of mutations relative to WT across different assays were log2 transformed to have comparable amplitudes (see materials and methods for details) and clustered based on Euclidian distance using Ward linkage. Variants with similar profiles of mutational effects across the assays are clustered together. Their magnitudes are color‐coded according to the scale bar. c) Structural context of lysine residues based on their mutational effects. The cross sections of representative relevant αSyn fibril polymorphs including MSA fold (6xyo), LB fold (8a9l), Polymorph 2a (6sst), and acetylated K80 mutant (9ptc). Lysine residues found in the structured fibril cores are highlighted in dark (K43, 45, 80) or light (K58, 60) brown, whereas those found in disordered regions in light blue. Residues with the most significant effects are highlighted in the sequence scheme bellow.

In order to gain a global overview into how individual KQ cluster mutations shape the αSyn assembly landscape, we grouped them based on their relative effects (compared to WT) observed in all our assays (Figure 6a,b). The results reveal that the mutational effects map onto the structural context of the lysine residues in known αSyn fibril structures. Specifically, mutations or residues found in the fibril cores (Figure 6c), K43+K45 (KQ4), K80 (KQ6), and to lesser degree K58+K60 (KQ5) have the largest and most distinct effects across all assays.

These observations are consistent with other studies showing that acetylation of lysines K43 and K80, or modifications of adjacent regions, significantly alter aggregation and seeding, identifying them as key modulators of αSyn aggregation [43, 49, 53, 92, 93]. Lysines 43 and 45 are located between previously identified critical regions P1 (residues 36–42) and P2 (residues 45–57) [53] and part of the β‐hairpin that was shown to be important for the fidelity of WT fibril elongation [93]. They are found in proximity of an unknown electron density within structures of fibrils isolated from brains of MSA [55] and PD [94] patients, that likely correspond to polyphosphate or other negatively charged molecules [92]. Mutation of these residues are examples of perturbations that significantly modify the free energy landscape of the WT protein, creating pathways and energy minima that are less likely to be sampled by WT monomer under the same experimental conditions (Figure 6c).

In contrast, mutations of lysines found in the disordered fuzzy coat of fibrils (KQ1‐3, KQ7, Figure 6b,c) primarily increase the energy barriers while overall maintaining WT‐like free energy landscape minima. Most notably, mutations of lysines K6, K10, K12 (KQ1) and K21, K23 (KQ2) lead to significantly slower aggregation across all types of aggregation assays tested here. Interestingly, mutation of K6, 10, and 12 to alanine was reported to have only minor, or even opposite, effects on aggregation [51]. We hypothesize that the more hydrophobic alanine substitutions reduce protein solubility and/or introduce new interactions that compensate for the loss of electrostatic interactions mediated by the original lysines. Reduced aggregation was observed previously for K6Q and K10Q [95], as well as for the acetylated K12 variant [43], highlighting the important roles of a positively charged N‐terminus in facilitating monomer interactions observed here. These appear to be highly specific, as demonstrated by the ability of the KQ1 and KQ2 variants to differentiate between PD and MSA brain‐derived samples in the low pH SAA.

### 3.5 Mutational Analysis as Quantitative Probe for Studying αSyn Assembly Energy Landscapes

The work presented here is an attempt to unify commonly used aggregation assays to assess the impact of sequence perturbations in a formalized and systematic manner. Our results demonstrate that mutational studies can quantitatively probe the αSyn energy landscape, provided they are carried out under well controlled conditions and complemented by structural, or morphological analysis to ensure meaningful and interpretable results. The clustering in Figure 6b provides a compelling framework for understanding how specific mutations, in this case those that alter electrostatic interactions, modulate the aggregation landscape.

However, expanding the mutational space will be essential for further validation and generalizing the observations made here. In this study, we selected mutations with the same chemistry to demonstrate the feasibility and scalability of such an approach. The scaled‐down purification protocol developed here allows to obtain ca 30 αSyn variants within 10 days in sufficient amount (1–3 milligrams) and purity (>95%) to perform all assays presented here (Figure S1). Extending the dataset by including (i) different mutations of the same residues (e.g., alanine, glutamate), (ii) mutations of negatively charged residues within the imperfect repeats (e.g. E‐to‐Q mutations), (iii) and mutations targeting different physio‐chemical properties (e.g., aromaticity, hydrophobicity) within specific sequence regions will provide more complex and comprehensive insights.

Moreover, the framework could be extended to other assays e.g. the formation of oligomers, and, importantly, cellular assays that would help bridge the observation of mutational effects in vitro to physiologically relevant environments. Together, these efforts will help clarify the pathways and sequence regions responsible for the transition from physiological to pathological αSyn conformations. They will also help identify in vitro assays with readouts that are directly translatable to biologically relevant outcomes that can be used for efficient and high‐throughput drug screening. Finally, a few variants appeared selective in seed amplification assays, making them promising candidates for further testing in the diagnosis of disease‐associated fibril conformations.

In conclusion, in our view, we demonstrate that a systematic and comprehensive approach to mutagenesis, such as all lysines to glutamines as done here, all but ensures the discovery of important effects (e.g., crucial sequence regions for mechanistic steps, suitable substrates for seed amplification assays), rather than having to rely on fortuitous discovery of individual effects through trial and error. We believe that the approach presented in our work presents a comprehensive framework within which we generate substantial novel understanding and where many previous studies and discoveries naturally fall into place.

## 4 Materials and Methods

### 4.1 Mutagenesis

Lysine‐to‐glutamine (KQ) variants were prepared starting from pET29a_αSyn WT plasmid using Golden gate mutagenesis protocol. The primers were ordered from TAG Copenhagen A/S (Denmark) and all chemicals, restriction enzymes and buffers from New England Biolabs (USA) unless stated otherwise. The protocol utilizes the set of primers provided in Table 1 and consists of (i) amplification (Table 2 and Table 3), (ii) purification, (iii) restriction/ligation, and (iv) transformation. Linear products of amplification were purified from an agarose gel after electrophoresis in 1% TAE agarose (120 V, 30 min) using GFX PCR DNA and Gel Band Purification Kit (Cytiva, USA). The Golden gate assembly mixture was prepared according to Table 2 and incubated at 37°C for 16 h. The reaction was stopped by 10 min incubation at 85°C. Residual template DNA was digested DpnI (1U, 1.5 h, 37°C incubation) that was then deactivated by heating (85°C, 10 min). The individual reactions were pooled together, cleaned using the GFX PCR DNA and Gel Band Purification Kit (Cytiva, USA), transformed into BL21(DE3) competent *E. coli*, which were plated on LB‐agar containing kanamycin as a selection marker (50 µg/mL), and incubated for 12 h at 37°C. Single colonies were transferred into a 96‐well plate containing 50 µL of TE buffer using a sterile toothpick and send for sequencing (Eurofins Genomics, Germany). The same colonies were simultaneously transferred to a 96‐DW plate containing 1 mL of LB (kanamycin) which was then used to create respective glycerol stocks. KQ cluster variants created in the first round of mutagenesis (together with single‐point mutants) were used as templates for preparation of the double‐cluster variants.

### 4.2 Protein Expression

#### 4.2.1 Large Scale Protein Expression and Purification

Wild type, KQ cluster variants, and familial mutants (E46K, H50Q, G51D, A53E) of αSyn were expressed in *E. coli* BL21 (DE3) cells transformed by the pET29a plasmid carrying the respective gene [96]. The transformed cells were used to inoculate 1 L of LB medium containing kanamycin (30 µg.mL^−1^) as selection marker. Following a 3‐hour incubation at 37°C (OD_600_∼0.6–0.8), protein expression was induced by the addition of IPTG (1 mM final concentration) and carried out for 4 h at 37°C. The cells were harvested by centrifugation (5,000×g, 20 min) and the resulting pellet resuspended in 20 mL of Tris buffer (10 mM Tris–HCl, 1 mM EDTA, pH 8.0) with 1 mM PMSF (phenylmethylsulfonyl fluoride). Cells were sonicated with a probe ultrasonicator for 8 min (10 s on time, 30 s off time, 12 rounds with 40% amplitude). 1 µL of commercial DNAse (Benzonase) was added to the cell lysate and the insoluble fraction was removed by centrifugation (20 000×g, 30 min at 4°C). The cell‐free extract was boiled for 20 min and the heat‐precipitated proteins removed by centrifugation (20 000×g for 20 min at 4°C). αSyn was precipitated by the addition of saturated (NH_4_)_2_SO_4_ (4 mL per 1 mL of supernatant). The solution was incubated on a rocking platform at 4°C for 15 min and then centrifuged (20 000×g, 20 min, 4°C) to obtain a protein pellet. The pellet was dissolved in 7 mL of 25 mM Tris–HCl pH 7.7 with 1 mM DTT. Protein was dialyzed against the same buffer for 16–18 h with a buffer exchange after 12 h of dialysis at 4°C. The dialyzed protein was then subjected to anion exchange chromatography (AEC) (HiTrap Q Hp 5 mL, Cytiva) followed by size exclusion chromatography (SEC) (HiLoad 16/600 Superdex 200 pg column, Cytiva). The monomeric fraction of αSyn eluted in 10 mM of sodium phosphate buffer (pH 7.4) was collected, and the protein concentration determined by UV‐absorption at 280 nm with theoretical molar extinction coefficients calculated from the protein sequence using ProtParam80 (Expasy, Switzerland).

### 4.3 Small‐Scale Protein Expression and Purification

The small‐scale expression of αSyn KQ variants (single‐point mutants, double KQ cluster variants) was carried out in 90 mL LB medium analogously to the large‐scale expression. Pellets from the harvested cells were resuspended in 100 mM MES, 750 mM NaCl, 1 mM EDTA, 1 mM PMSF pH 7 and heated to 80°C for 30 min. Next, acetic acid (c_final_ = 1% v/v) and streptomycin (c_final_ = 1% w/v) were added, and the insoluble fraction was removed by centrifugation (20 000×g, 20 min, 4°C). The resulting cell‐free extract was precipitated by the addition of saturated (NH_4_)_2_SO_4_ (4 mL per 1 mL of supernatant). The solution was incubated on a rocking platform at 4°C overnight and then centrifuged (20 000×g, 30 min, 4°C). The resulting protein pellet was dissolved in 4 mL of 10 mM NaP buffer pH 7.4 and dialyzed twice to the same buffer. Proteins were purified by AEC using AcroPrep 96‐well filter plates (Cytiva, USA) and Multi‐well Plate Vacuum Manifold (Cytiva, USA). Each well was loaded with 350 µL of Nuvia HP‐Q strong anion exchange resin (Bio‐Rad, USA). Resins were washed by 10 mM NaP buffer pH 7.4 before samples were applied (1 mL/well, 4 wells/sample). Unbound proteins were washed with 5 × 0.6 mL of 10 mM NaP buffer and 5 × 0.6 mL of 10 mM NaP buffer with 100 mM NaCl. Single‐point mutants, KQ clusters variants, and double KQ cluster variants were eluted by 10 mM NaP buffer supplemented with 250, 300, or 350 mM NaCl, respectively. Proteins were concentrated and buffer‐exchanged using Amicon Ultra Centrifugal Filters with 3 kDa Mw cut‐off (Merck, USA), aliquoted, flash‐frozen in liquid nitrogen, and stored at −80°C. Their concentration, purity and size distribution were determined using UV‐absorbance (ε_280_ = 5,960 cm^−1^M^−1^), SDS‐PAGE analysis and flow‐induced dispersion (FIDA) analysis.

### 4.4 Fibril Preparation

100 or 200 µM αSyn WT or KQ cluster monomers were buffer exchanged into the corresponding assembly conditions (Table 4). After the incubation, fibrils were pelleted by centrifugation (16,000 x g, 60 min, 25°C) and the supernatant was carefully removed. The residual monomer concentration was quantified from the isolated soluble fraction using UV‐absorbance, SDS‐PAGE and FIDA. The fibrils were resuspended in their respective assembly buffers to final concentration of 100 or 200 µM (in monomer equivalents), flash‐frozen in liquid nitrogen and stored at −20°C. Prior to the experiments (seeding assays, chemical depolymerization), fibrils were thawed and sonicated using an ultrasonic probe (Hielscher UP200St). Sonication was carried out in repeating 1s‐pulses of 100% amplitude separated by 1 s pauses for 4 min (2 min total sonication time).

### 4.5 Thioflavin T Assays

All ThT kinetic measurements were carried out using the FLUOstar Omega plate reader (BMG, Germany) using 440/488 excitation and emission and bottom reading. All reactions contained 50 µM ThT (final concentration) and were carried out at 37°C in triplicates with reaction volumes of 100 µL (large scale, 96‐well plates) or 15 µL (small‐scale, 384‐well plates) per well. The conditions of specific ThT assays are provided in Table 5.

#### 4.5.1 De Novo Aggregation Assays

Protein samples were buffer exchanged into the assay conditions (Table 5), diluted to the final protein concentrations and supplemented by 50 µM ThT. Resulting kinetic curves were fitted to the sigmoidal function described by Equation (M1):

(M1) $$y=y_{0}+A/\left(1+\exp\left(-k\left(t-t_{0.5}\right)\right)\right)$$

where y_0_ is the pre‐transition baseline, A is the signal amplitude, k is the apparent growth rate, and t_0.5_ is the midpoint of the transition, i.e., half‐time [59].

The theoretical net charges of different variants at varying pH conditions were calculated using the Henderson‐Hasselbach equation based on the protein sequence as

(M2) $$\sum_{i=1}^{negative}-1/\left(1+10^{pK_{n}-pH}\right)+\sum_{j=1}^{positive}1/\left(1+10^{-pK_{n}+pH}\right)$$

where the pK_n_ and pK_p_ are dissociation constants of negatively and positively charged amino acid groups, respectively.

#### 4.5.2 Seeded Aggregation Assay

A dilution series of the αSyn variants was prepared in 5–80 µM monomer range and sonicated seeds were added (final concentration of 2.5 µM) just prior the measurement. A control reaction without ThT containing 40 or 80 µM of monomer was included for each series and used for quantification of the residual monomer concentration at the end of the reaction. A linear curve was fitted to the first 2.5 h of the data and resulting slopes plotted against the initial monomer concentration [M]_0_). The apparent elongation rate constants were extracted as the slopes in the linear data range of the plots [M]_0_ ∼ 0–40 µM). To cancel out the contribution of the number of seeding‐competent fibril ends [S], the effect of mutations was related to the elongation of WT measured on the same plate with the same batch of seeds according to Equation (M3).

(M3) $$\begin{array}{cc} \Delta \Delta G^{‡} & =\Delta G^{‡}\left(mut\right)-\Delta G^{‡}\left(WT\right)\\ & =-RT\;In\left(k_{+}^{mut}\left[S\right]\right)+RT\;In\left(k_{+}^{wt}\left[S\right]\right)\\ & =-RT\;In\left(k_{+}^{mut}/k_{+}^{wt}\right) \end{array}$$

where ΔG^ǂ^ is the Gibbs activation energy of elongation, R the universal gas constant, T the thermodynamic temperature, and *k*_+_ the microscopic elongation rate constant of wild type (wt) or mutant (mut).

In cases where saturation elongation was observed, initial rates were fitted to Eq (M4).

(M4) $$\nu =v_{\max}\left[M\right]/\left(\left[M\right]+K_{e}\right)$$

where v and v_max_ is the observed and maximum rate, respectively, *K*_e_ is the elongation saturation constant, and [M] is the monomer concentration [97].

#### 4.5.3 Seed Amplification Assay at Low pH

WT or mutant monomers (10 µM) were brought to the assay conditions by mixing from a high protein concentration stock. Sonicated WT‐Fm or WT‐Ri fibrils were added to the samples to final concentrations of 0, 0.001, or 1 µM. The kinetic curves in the presence of 1 nM were fitted by Equation (M1) to extract the aggregation half‐times used for further analyses.

Brain samples (Table 6) were acquired from the Bispebjerg Brain Bank at Bispebjerg‐Frederiksberg Hospital (University Hospital of Copenhagen, Denmark; Ethical approval: j.no.: H‐15016232, data protection agency: j.no.: P‐2020‐937, Table 6). Brain tissue homogenates were prepared as follows: Approximately 50 mg of tissue samples were homogenized using a bead homogenizer (Precellys, Bertin Technologies) with 2 cycles of 45 s at 4,600 rpm in buffer containing 1x dPBS (Gibco), 1x HALT protease and phosphatase inhibitor cocktail (cat no.: 78444, Thermo Scientific) to a final concentration of 10% w/v. Aliquoted homogenates were stored at −80°C for further use.

**TABLE 6:** Overview of the patients derived samples used for seed amplification assay.

Group	Number	Age (years)	Sex (Male/Female)	Disease duration (years)	PMI (hours)	Subtype (P/C)
MSA	3	65.0 (7.8) [56‐70]	3/0	8.0 (1.0) [7‐9]	36.0 (19.1) [24‐58]	3/0
PD	3	87.3 (7.1) [81–95]	2/1	9.3 (2.5) [7‐12]	53.0 (28.7) [24‐96]	—
Control	3	81.0 (11.8) [68‐91]	1/2	—	48.0 (41.6) [24‐96]	—
p‐value	—	p = 0.168^§^	p = 0.679^#^	p = 0.800^&^	p = 0.668^§^	—

PMI: Post mortem interval (hours). Subtype: P = MSA parkinsonism subtype, C = MSA cerebellar subtype. MSA: multiple system atrophy. PD: Parkinson's disease. §: Kruskall‐Wallis test with Dunn's multiple comparison test. #: Fisher's exact test. &: Mann‐Whitney U test. Mean (SD) [range].

The plate assay was carried out as described in [85]. A buffer mix of 200 mM citric acid pH 2.5, Na_2_SO_4_ (2.5 M), ThT (1 mM), and NaN_3_ (10% w/v) was prepared in 14:7:3.5:0.5 ratio and filtered with a sterile syringe filter. Solution of monomeric αSyn variants was filtered through 100 kDa centrifugal filter unit, (Amicon) at 3,000 x g, 5 mins at 25°C. Membrane was washed 10 mM NaPi (pH 7.4) using the same protocol, the concentration of αSyn in the flow‐through determined using NanoDrop (ThermoFisher, USA) and adjusted to 20 µM using the same buffer. Brain homogenates were diluted 10^−3^ in PBS with human serum albumin (1:200; Fujifilm Biosciences, cat#9988, lot#0000017008). A master mix was prepared by combining 35 µL of buffer mix with 25 µL of protein solution in a low binding tube, carefully mixed with 10 µL of sample (PD, MSA brain homogenate) or control (healthy brain, PBS) (Table 6), and transferred to the well plate at 15 µL aliquots. Each solution in the well was carefully topped by 4 µL of the 1% FluoSurf‐C Neat surfactant in Fluo‐Oil 40.

### 4.6 Weighted Least‐Square Regression of the Data From Aggregation Assays

The data from unseeded experiments, elongation experiments with WT seeds at neutral pH, and mildly seeded experiments at low pH were compiled into three datasets. Variants were one‐hot encoded at the single‐mutation (elongation) or KQ‐cluster (unseeded and low‐pH) level, and each data point was parameterized by the number of mutations, pH, ionic strength, and net charge (Equation M2) derived from the experimental conditions. Experimental replicates were grouped by variant and fibril type to calculate mean ΔΔG values and corresponding standard errors of the mean (SEM), with each group assigned an inverse‐variance weight (1/SEM^2^). For the unseeded and low‐pH datasets, mean and relative standard deviation (SDrel = SD/mean) of log‐transformed half‐times from each triplicate measurement were used as a single data point and weights (1/SDrel), respectively. The noise level was estimated from the average correlation between replicate measurements, computed using Fisher's z‐transformed mean. Weighted least squares (WLS) regression and statistical analyses were performed in Python (v3.11) using statsmodels (v0.14).

### 4.7 Quantification of Residual Monomer

#### 4.7.1 UV Absorbance

Samples without ThT were withdrawn from the plate and centrifuged to pellet down the fibrils (16,000 x g, 60 min, 25°C). The concentration of monomer was determined by UV absorbance using NanoDrop (ThermoFisher, USA) and the extinction coefficient of αSyn (ε_280_ = 5,960 cm^− 1^M^−1^) calculated from the sequence using the Expasy webserver.

#### 4.7.2 Flow‐Induced Dispersion Analysis (FIDA)

The oligomeric state analysis of the supernatant and monomer quantification were further determined using the FIDA1 instrument (FidaBio, Denmark). Samples were analyzed using the method provided in Table 7.

**TABLE 7:** FIDA method used for analysis of soluble αSyn fraction.

Step	Component	Time (s)	Pressure (mbar)	Temperature (°C)
Wash 1	1 M NaOH	45	3500	25
Wash 2	Water	45	3500	25
Equilibration	Buffer	40	3500	25
Sample	Protein	20	75	25
Measurement	Buffer	75	1500	25

The monomer concentration was quantified from the areas under the peak (obtained by fitting a Gaussian function to the Taylorgrams by the in‐built software) using a calibration curve of known monomer concentrations.

#### 4.7.3 SDS‐PAGE Analysis

The samples were collected from the assay plate and centrifuged to pellet down the fibrils (16,000 x g, 60 min, 25°C). The supernatant was mixed with the NuPAGE LDS Sample Buffer (ThermoFisher) in a 1 to 1 ratio and applied to NuPAGE Bis‐Tris Mini Protein Gels, 4–12% (ThermoFisher). Calibration samples of SEC‐isolated αSyn monomer of known concentrations were prepared in the same way. The electrophoresis was carried out at constant 200 V for 35 min, followed by staining using InstantStain Coomassie Stain (Kem‐en‐tec‐nordic). Upon destaining in distilled water, the gels were imaged using ChemiDoc imaging system (BioRad), and the intensity of bands corresponding to αSyn was analyzed using Image Lab software (BioRad). The concentration of residual monomer was calculated based on the calibration curve made using the monomer standards of known concentrations.

### 4.8 Quartz Crystal Microbalance Analysis of Fibril Growth

Elongation of WT or KQ fibrils were measured by their immobilization on a QCM sensor (Biolin Scientific, Gothenburg, Sweden) and measuring changes in mass upon subsequent incubation with WT or KQ monomer solution [98]. Sonicated fibrils (65 µL, 100 µM monomer equivalent) were mixed with 10 µL of 1 mg.mL^−1^ of Traut's reagent (2‐Iminothiolane, ThermoFisher) and spotted on the QCM sensor following 1 h incubation at room temperature. Next, the solution was pipetted out and the chip surface was blocked by addition of 1% mPEG and incubation for 30 min. The sensor with immobilized fibrils was then thoroughly washed by miliQ H_2_O and dried under gentle nitrogen stream. The measurements were performed with a QSense Pro QCM‐D instrument (Biolin Scientific, Gothenburg, Sweden) by measuring the elongation rate as a change in the resonant frequency over time. The sample chamber equilibrated to 37°C was filled automatically by 3 cell volumes (60 µL) of WT monomer (50 µM) and the measurement proceeded until a stable linear slope was achieved. Next, the sensor was cleaned using buffer (50 mM Tris‐HCl 150 mM KCl pH 7.4) and mutant monomeric solution was injected until a stable slope was achieved. The elongated rates of WT and mutant variants were measured as the slopes of the third overtone frequency after the first and second injections and used to calculate ΔΔG^ǂ^ according to the Equation (M3). For KQ fibrils, the sequence of WT and KQ mutant monomer addition was reversed.

### 4.9 AFM Analysis of the Fibrils

Fibrils were diluted to 2.5 µM monomer equivalent concentration and 20 µL of the solution was deposited onto freshly cleaved mica substrates. Following 2 min of incubation, the substrates were cleaned extensively with miliQ water and dried under nitrogen gas flow. All fibrils were imaged in tapping mode in air using a DriveAFM (Nanosurf, Liestal, Switzerland) using PPP‐NCLAuD cantilevers (Nanosensors, Neuchatel, Switzerland). The amyloid fibrils were characterized by their apparent twist (assuming 2_1_ helical symmetry as described for Fm polymorphs in [68] and height extracted using an automated python script [54, 71].

### 4.10 Transmission Electron Microscopy of KQ4 and KQ6 Fibrils

Samples were prepared on a glow discharged, formvar/carbon‐coated 400 mesh grid for 20 s. The grids were then washed with two drops of double‐distilled water and stained twice with 2% uranyl acetate. Excess stain was blotted, and the grids were air‐dried for 30 min before imaging. A 200 kV Tecnai T20 G2 electron microscope (FEI, USA) was used to analyze the fibrils. Images were captured with a TVIPS XF415 CMOS 4K camera using TVIPS EMplify v0.4.5 software.

### 4.11 Thermodynamic Stability of Fibrils

The thermodynamic stability of WT and mutant fibrils was measured in 50 mM Tris‐HCl 150 mM KCl buffer pH 7.4 using chemical depolymerization and FIDA analysis of residual monomer as described in [69]. In short, sonicated fibrils (40 µM monomer equivalent) were incubated in series of buffers containing increasing concentrations of urea (0‐5 M) for 3 days at 25°C. Samples were analyzed by FIDA using the method described in Table 7. The monomer concentration was extracted from the elution profiles after correction for the viscosity at different urea concentrations as previously described [69]. The chemical depolymerization curves were analyzed using NumPyro to sample posterior distributions of the isodesmic model (Equation M5) parameters using the No U‐Turn Sampler (NUTS) [70, 71, 99].

(M5) $$\begin{array}{cc} A & =2/\left(1+2M\exp\left(-\Delta G+mD\right)/RT\right)\\ & \;+\sqrt{1+4M\exp\left(-\left(\Delta G+mD\right)/RT\right)} \end{array}$$

where A is the area under the Gaussian peak from FIDA, M is the protein concentration in monomer equivalents, ΔG is the thermodynamic stability, m is the m‐value, R the universal gas constant, and T is the temperature.

### 4.12 FoldX Analysis of Mutational Changes on Fibril Stability

FoldX calculations were performed on a curated set of aSyn fibril structures selected from the Amyloid Atlas (v2024) [72]. We curated the full set of aSyn structures by excluding (i) structures formed from conditions significantly different from physiological conditions used in this study, (ii) containing other compounds – such as lipids – or (iii) formed from mutant variants. Subsequently, the structures were aligned and manually investigated for similarity and structures with extensive overlap were removed to minimize bias from a single motif being represented multiple times. This curated set of 47 structures (Table S8) represent the two major structure families recently identified in a meta‐analysis of the full aSyn structure library, as well as the largest of the minor groups [100].

FoldX calculations were performed using the FoldX4 suite [91] as described in [54]. Briefly, before modelling any mutations in the structures, all PDB files were repaired using the REPAIRPDB command. Subsequent commands were only performed on the repaired structures. The ΔΔG upon mutagenesis was calculated using the BUILDMODEL command, ensuring that the mutation was introduced in all chains of the PDB file. The total ΔΔG is divided by the number of chains in the structure to evaluate a per chain ΔΔG. This was done for all possible single KQ mutations in the curated structure set.

### 4.13 Clustering of Mutational Effects

Fibril stability, ΔΔG^ǂ^ values on KQ seeds, and cluster‐specific coefficients from WLS regression of data from unseeded experiments, elongation experiments on WT at neutral pH, and mildly seeded experiments at low pH were log_2_‐transformed. Biclustering of the resulting values was performed using seaborn.clustermap (Python 3.11) with hierarchical clustering applied to both assays and KQ variants, using the default settings of Euclidean distance and average linkage.

### 4.14 Molecular Dynamics Simulations of αSyn Monomers

All simulations were prepared and executed with the CALVADOS Python interface and through the publicly available Google Colab [67]. A single protein chain was simulated with both N‐ and C‐terminal charges. The initial configuration was centered in a cubic box with a side length of 40 nm. Simulations were performed at 310.15 K, pH 7.5, and a range of ionic strengths (0, 5, 50, and 150 mM). Trajectories were saved every 100,000 integration steps (equivalent to 1 ns per saved frame). A total of 1000 frames were saved per replicate, corresponding to 100,000,000 integration steps and an aggregate production length of 1 µs. From each trajectory, ensemble observables including the radius of gyration (R_g_), end‐to‐end distance (R_ee_), Flory scaling parameter (ν), and energy interaction map were computed automatically. Reported values represent averages across three independent replicates for each condition. Residue–residue contacts were calculated using a 9 Å cutoff applied to coarse‐grained bead distances, excluding pairs with 𝑖−𝑗 ≤ 2 to remove bonded and next‐nearest neighbor contributions. Contact probabilities and per‐residue profiles were averaged across all three replicates. To connect simulations to experiment, R_g_ values were correlated with the aggregation half‐times measured experimentally.

## Author Contributions

A.K.B. supervised the work. A.K., S.F., R.K.N., and A.K.B. conceptualized the work. R.K.N., S.F., and A.K. designed the mutant primers, A.K. designed and carried out the experiments, analyzed the data, prepared the graphics, and wrote the manuscript. A.K. and S.F., carried out the seed amplification assay experiments, J.A.L and A.K. performed the QCM measurements, A.K., F.S., and C.F., expressed and purified the mutants, H.M.B. and A.S. carried out TEM analysis of the KQ fibrils, R.K.N. and C.F. wrote the python code for the analysis of urea depolymerization experiments. C.F. helped with preparation of the graphics. J.F. and S.A. prepared the samples for seed amplification assay. All authors contributed to the preparation of the manuscript and agree with its content.

## Conflicts of Interest

The authors declare no conflicts of interest.

## Supporting information




**Supporting File**: advs77643‐sup‐0001‐SuppMat.docx.

## Data Availability

The raw data associated with this study are available at Zenodo repository. https://doi.org/10.5281/zenodo.17591639.
