# Supplementary material for: Probing the Energy Landscape of α‐Synuclein Amyloid Fibril Formation by Systematic K‐to‐Q Mutagenesis
Source: Adv Sci (Weinh). 2026 Sep 27:e77643. Online ahead of print. doi: 10.1002/advs.77643 (PMC13616359; doi:10.1002/advs.77643)
Supplement: Supplementary file 1 [file ADVS-9999-e77643-s001.docx]

# Supplementary Information

# Probing the energy landscape of α-Synuclein amyloid fibril formation by systematic K-to-Q mutagenesis.

Antonin Kunka^[a]^, Azad Farzadfard^[a]^, Jacob Aunstrup Larsen^[a]^, Rasmus Krogh Norrild^[a]^, Federica Saraceno^[a]^, Celia Fricke^[a]^, Hossein Mohammad-Beigi^[a]^, Ahmed Sadek^[b]^, Jonas Folke^[c]^, Susana Aznar^[c]^, Alexander K. Buell^#[a]^

[a]	Department of Biotechnology and Biomedicine

Technical University of Denmark

Søltofts Plads, Building 227, 2800 Kgs. Lyngby, Denmark

[b]	Department of Biotechnology and Bioengineering

Ecole polytechnique fédérale de Lausanne EPFL

[c]	Centre for Neuroscience and Stereology, Department of Neurology, Copenhagen University Hospital, Bispebjerg and Frederiksberg Hospital, Nielsine Nielsens Vej 6B, Entrance 11B, 2. Floor, 2400 Copenhagen, Denmark.

\# E-mail: [alebu@dtu.dk](mailto:alebu@dtu.dk)

**Table of contents**

Supplementary Figures 1-21	2-24

Supplementary Tables 1-8	25-35

References	36

# Supplementary Figures

**Supplementary Figure 1. Small scale purification of mutant αSyn variants. a) SDS-PAGE analysis** of single-point KQ mutants after (1) thermal lysis and centrifugation of insoluble fraction, (2) precipitation by ammonium sulphate and subsequent centrifugation, and (3) anion exchange chromatography (AEC) in plate format. The control αSynWT monomer sample purified using standard protocol including size-exclusion is indicated by the star. **B) SDS-PAGE analysis** of double-point KQ mutants, KQ cluster, and double-KQ cluster variants after AEC. **C) Hydrodynamic radii** of the purified mutants determined using flow-induced dispersion analysis (FIDA). **D) Total yield** of all variants from 90 mL of LB cultures after AEC purification.

**Supplementary Figure 2. De novo aggregation of KQ cluster variants** monitored by ThT Fluorescence with varying **(a)** initial protein concentration (20, 40, 60,100 μM), **(b)** pH (4.6, 5.4, 6.2, 7.4), and **(c)** concentration of KCl (0, 50, 150, 300 mM). The color gradients presented on the right sides of the graphs indicate the increasing value of the variable parameter for each mutant. **(d)** Monomer conversion to fibril calculated from the residual monomer analysis of soluble fraction withdrawn from the wells using SDS-PAGE.

**Supplementary Figure 3. Analysis of the residuals** from the regression model of aggregation half-times show in Figure 2. **(a) Quantile–quantile (QQ) plot of model residuals.** The plot compares the distribution of standardized residuals from the final model against the theoretical quantiles of a normal distribution. Points falling along the red line indicate approximate normality. **(b) Residuals by assay type.** The points correspond to residuals from the model (observed-predicted) grouped by the assay type. The box represents the interquartile range (IQR), the horizontal line shows the median, whiskers extend to 1.5×IQR, and the red dashed line marks the perfect model fit (zero residuals). **(c Residuals by variant.** The points correspond to residuals from the model (observed-predicted) group by the assay type. The box represents the interquartile range (IQR), the horizontal line shows the median, whiskers extend to 1.5×IQR, and the red dashed line marks the perfect model fit (zero residuals).

**Supplementary Figure 4. Molecular dynamics simulations of αSyn variants. a) Interaction energy maps** derived from simulations of single protein chains at 0 (top) or 150 mM (bottom) ionic strength. The interaction strength is color-coded according to the legend. **b) Sequence profiles of average intra-chain residue contacts** for WT and KQ cluster variants, excluding the neighbouring residues. The contact cut-off value was 9 Å. The plots correspond to the average from three simulation replicates and SE. The monomer secondary structure within fibril plane is provided on top for illustration. **c) Global scaling properties** derived from the MD simulations at varying ionic strengths. The values correspond to means ± SE from three simulation replicates. The red asterisk at the bottom graph denotes values resulting from poor fitting of the R_ij_=R_0_|i-j|^3^ power law. **d) Correlations between experimental aggregation half-times and MD-derived compactness** (end-to-end chain distance). The solid lines correspond to linear fits to the data from individual mutants. Dashed line represents a global linear fit

**Supplementary Figure 5. Chemical depolymerization of fibrils formed by WT and KQ cluster variants of αSyn. (a) and (b) Chemical depolymerization of different fibril batches.** Data points (area under the curve obtained from FIDA measurements corresponding to monomeric αSyn) and their fits to the isodesmic model using MCMC sampling of solutions (thin lines correspond to the 100 random solutions out of 2000 total, best fit is shown in bold). **(c) Correlations of the m-values and ΔG** obtained from the fitting. The possible solution space is represented by kernel density estimation color-coded according to the mutants.

**Supplementary Figure 6. Aggregation kinetics using KQ seeds. (a) Aggregation kinetics monitored by ThT.** The mutant (left column) or WT (right column) monomers were seeded by 2.5 μM sonicated KQ fibrils. The color-scheme highlights different initial concentrations of monomers provided in the legend below. **(b) Aggregation kinetics monitored using quartz crystal microbalance (QCM).** The KQ seeds immobilized on a QCM sensor were first elongated by their respective monomer (homogeneous elongation, coloured boxes, followed by elongation by WT monomer (grey boxes), with thorough washing steps in between. The elongation rates of the mutant and WT were obtained by fitting the linear part of the change in the frequency of 3^rd^ harmonic overtone (black line) from the first and second injection, respectively. **(c) Residual monomer analysis.** Soluble monomer at the end of aggregation reaction was quantified either by SDS-PAGE analysis or UV absorbance (ThT free samples, diamonds). The y-axis represents percentage of initial monomer concentration converted to fibrils. The grey bars and error bars represent mean ± SE from three independent measurements.

**Supplementary Figure 7. Fibril morphology of WT and mutant fibrils.** AFM images of the fibrils formed by aggregation of the αSyn monomers in the presence of pre-formed WT or KQ fibrils. The fibril heights in nm are indicated by the color-coding. The scale bars represent 2 μm.

**Supplementary Figure 8. Representative TEM images of (a) KQ4 and (b) KQ6 fibrils.** The scale bars in a and b correspond to 500 and 100 nm, respectively.

**Supplementary Figure 9. Reproducibility of the seeded aggregation assays. (a) Raw data from ThT aggregation experiments** using WT monomer and pre-formed WT Fm seeds. The initial monomer concentrations used are indicated in the legend. (**b) Initial rate analysis.** The initial rates were calculated from the data shown in (a) in the time range of 0 – 1 h (blue), 0 – 2.5 h (green), and 0 - 5 h (red). The fits of the initial rates as a function of initial monomer concentration to the equation describing saturating elongation are shown as dotted lines. The saturation concentrations (K_e_) were fitted globally whilst the maximal rates were treated as free parameters within each dataset.

**Supplementary Figure 10. Seeded aggregation kinetics of single-point mutants on WT Fm seeds** followed using ThT kinetics. The initial rates as a function of initial concentration were used to derive ΔΔG^ǂ^ of elongation (one of the points in Figure 4c. The initial monomer concentrations used in the experiments are shown in the legend.

**Supplementary Figure 11. Example of aggregation kinetics of KQ clusters** seeded by WT-Fm (2.5 μM monomer equivalent). The initial monomer concentrations used in the experiments are shown in the legend.

**Supplementary Figure 12. Seeded aggregation kinetics of double KQ clusters on WT-Fm seeds** (2.5 μM monomer equivalent) carried out in three individual datasets shown in a, b, and c. The initial monomer concentrations used in the experiments are shown in the legend.

**Supplementary Figure 13. Analysis of residual soluble monomer concentration** at the end of the aggregation assays seeded by WT Fm polymorph. The points corresponding to the fraction of monomers converted to fibrils were obtained by SDS-PAGE, UV absorbance, and FIDA analyses or their combination from each elongation datasets with **A)** single-point mutants, **B)** KQ cluster variants, and **C)** double KQ cluster variants. The bar and error bar represent mean + SE, respectively.

**Supplementary Figure 14. Modelling of relative growth rates (ΔΔG^ǂ^) on WT-Fm by linear regression** as a function of number of mutations (**a**), or mean cluster contributions (**b**). Individual points correspond to the mean values from Figure 4c with 1:1 line depicted in red. Residuals (observed-predicted) are shown on top.

**Supplementary Figure 15. Amplification of Fm seed at low pH.** The data show raw data from two datasets with **(a)** single cluster and **(b)** double cluster variants. The corresponding WT references are shown in **c**. The concentration of seeds was 0 (grey), 1 nM (light green), and 1 μM (dark green).

**Supplementary Figure 16. Amplification of Ri seed at low pH.** The data show raw data from two datasets with **(a)** single cluster and **(b)** double cluster variants. The corresponding WT references are shown in **c**. The concentration of seeds was 0 (grey), 1 nM (light purple), and 1 μM (dark purple).

**Supplementary Figure 17. Raw data from seed amplification assay** with brain homogenates of patients with MSA (green), PD (orange), healthy controls (CTRL, light grey), or negative controls (NEG, dark gray).

**Supplementary Figure 18. Example of small-scale (384-well plate) de novo aggregation of KQ cluster variants** monitored by ThT Fluorescence with varying **(a)** initial protein concentration (5, 10, 20, 40, 80 μM), **(b)** pH (4.6, 5.4, 6.2, 7.4), and **(c)** concentration of KCl (0, 50, 100, 200 mM). For WT in a, the concentrations are 6.25, 12.5, 25, 50, and 100 μM. The color gradients indicate the increasing value of the variable parameter for each mutant. **(d)** Aggregation half-times extracted from fitting the data by equation M1. Points depict the individual values whilst bars represent the average (n=3).

**Supplementary Figure 19. Comparison of results from de novo aggregation assays** carried out in large (96-well) and small (384-well plates) scale. **a)** Weighted linear regression of log-transformed half-times from the small-scale de novo aggregation assay using model described by by Eq. 1 with β_m_=0. Each point corresponds to the mean half-time from a triplicate measurement (Supplementary File 1), dashed red line indicates 1:1 line. Residuals (observed-predicted) are shown on top. **b)** Variant specific coefficients derived from the model in a. Error bars indicate ± 95% confidence intervals. Asterisks denote levels of statistical significance: * p < 0.05; ** p < 0.01; *** p < 0.001, ns – not significant. **c)** Decomposition of global coefficients from the model in based on the explained variance. The model prediction (red line) is overlayed with the observed data (black circles) normalized for all but one model parameter. **d)** Correlation between half-times measured in the small (96-well) scale and large (384-well) plate format. Values from concentration, pH, and salt assays are depicted as diamonds, circles, and triangles, respectively. Only values measured under comparable conditions were used. Identity (1:1) is shown as dashed line. **e)** Correlation between all (e) or variant-specific **(f)** model coefficients (Eq. 1 with β_m_=0) from fitting of the small and large scale datasets (Supplementary File 1). N denotes the number of parameters and r the Pearson’s correlation coefficient (Supplementary Tables 2 and 7).

**Supplementary Figure 20. De novo aggregation of αSyn familiar mutants** monitored by ThT Fluorescence with varying **(a)** initial protein concentration (20, 40, 60,100 μM), **(b)** pH (4.6, 5.4, 6.2, 7.4), and **(c)** concentration of KCl (0, 50, 150, 300 mM). The color gradients presented on the right sides of the graphs indicate the increasing value of the variable parameter for each mutant. **(d)** Aggregation half-times extracted from fitting the data by equation M1. Points depict the individual values whilst bars represent the average (n=3). **(e)** Monomer conversion to fibril calculated from the residual monomer analysis of soluble fraction withdrawn from the wells using SDS-PAGE. **(f)** Prediction of aggregation half-times for the familiar mutants based on the coefficients derived from fitting the general scaling model (eq. 2) to the KQ dataset (Figure 2b left, Supplementary Table 2). Gray points correspond to the values from the KQ dataset, data from familiar mutants are color-coded according to the legend. Red dashed line is the identity (1:1). Residuals (observed-predicted) are shown on top). **g)** Residuals from the model in f grouped by the familiar mutant. Average values are shown as line. Asterisks denote levels of statistical significance of the residuals from zero (red dashed line) : * p < 0.05; ** p < 0.01; *** p < 0.001, ns – not significant.

**Supplementary Figure 21. (a) Fraction of residues resolved in known αSyn fibril structures.** The data were taken from curated set of fibril structures from Amyloid Atlas.(1) **(b) In silico predictions of fibril stability changes** upon mutation of lysines to glutamine using FoldX.(2) The analysis was carried out on 47 representative fibril structures from Amyloid Atlas database (Supplementary table 8). (1,3)

## Supplementary Tables

**Supplementary Table 1. Overview of the αSyn variants and assays used in this study.** The variant studied in a given assay is indicated by x. Conventional naming of single-point and double-point mutants is used throughout the manuscript (e.g., K6Q). Mutation K80Q is designated as variant KQ6 for consistency with the other cluster variants. # - number of mutations, [c] – de novo aggregation with varying protein concentration, I - – de novo aggregation with varying salt, pH - – de novo aggregation with varying pH, St – Fibril stability, A – AFM analysis of fibrils, E – elongation on WT fibrils, S – Seed amplification assay

| **Variant name** | **#** | **K residues mutated to Q** | **Experimental assays** | | | | | | | |  |
| --- | --- | --- | --- | --- | --- | --- | --- | --- | --- | --- | --- |
|  |  |  | **[c]** | **I** | **pH** | **St** | **A** | **E** | **S** | | |
| Single-point mutants | | | | | | | | | |  |  |
|  | 1 | 6 |  |  | x |  |  | x |  | | |
|  | 1 | 10 |  |  | x |  |  | x |  | | |
|  | 1 | 12 |  |  |  |  |  | x |  | | |
|  | 1 | 21 |  |  |  |  |  | x |  | | |
|  | 1 | 23 |  |  |  |  |  | x |  | | |
|  | 1 | 32 |  |  |  |  |  | x |  | | |
|  | 1 | 34 |  |  |  |  |  | x |  | | |
|  | 1 | 43 |  |  |  |  |  | x |  | | |
|  | 1 | 45 |  |  |  |  |  | x |  | | |
|  | 1 | 58 |  |  |  |  |  | x |  | | |
|  | 1 | 60 |  |  |  |  |  | x |  | | |
| KQ6 | 1 | 80 | x | x | x | x | x | x | x | | |
|  | 1 | 96 |  |  | x |  |  | x |  | | |
|  | 1 | 97 |  |  |  |  |  | x |  | | |
|  | 1 | 102 |  |  |  |  |  | x |  | | |
| Single cluster mutant variants | | | | | | | | | |  |  |
| KQ1 | 3 | 6+10+12 | x | x | x | x | x | x | x | | |
| KQ2 | 2 | 21+23 | x | x | x | x | x | x | x | | |
| KQ3 | 2 | 32+34 | x | x | x | x | x | x | x | | |
| KQ4 | 2 | 43+45 | x | x | x | x | x | x | x | | |
| KQ5 | 2 | 58+60 | x | x | x | x | x | x | x | | |
| KQ7 | 3 | 96+97+102 | x | x | x | x | x | x | x | | |
| Double cluster mutant variants | | | | | | | | | |  |  |
| KQ1+2 | 5 | 6+10+12+21+23 |  |  |  |  |  | x | x | | |
| KQ1+3 | 5 | 6+10+12+32+34 |  |  |  |  |  | x | x | | |
| KQ1+4 | 5 | 6+10+12+43+45 |  |  |  |  |  | x | x | | |
| KQ1+5 | 5 | 6+10+12+58+60 |  |  |  |  |  | x | x | | |
| KQ1+6 | 4 | 6+10+12+80 |  |  |  |  |  | x | x | | |
| KQ1+7 | 6 | 6+10+12+96+97+102 |  |  |  |  |  | x | x | | |
| KQ2+3 | 4 | 21+23+32+34 |  |  |  |  |  | x | x | | |
| KQ2+4 | 4 | 21+23+43+45 |  |  |  |  |  | x |  | | |
| KQ2+5 | 4 | 21+23+58+60 |  |  |  |  |  | x | x | | |
| KQ2+6 | 3 | 21+23+80 |  |  |  |  |  |  | x | | |
| KQ2+7 | 5 | 21+23+96+97+102 |  |  |  |  |  | x | x | | |
| KQ3+5 | 4 | 32+34+58+60 |  |  |  |  |  | x | x | | |
| KQ3+6 | 3 | 32+34+80 |  |  |  |  |  | x | x | | |
| KQ3+7 | 5 | 32+34+96+97+102 |  |  |  |  |  | x | x | | |
| KQ4+5 | 4 | 43+45+58+60 |  |  |  |  |  | x | x | | |
| KQ4+6 | 3 | 43+45+80 |  |  |  |  |  | x | x | | |
| KQ4+7 | 5 | 43+45+96+97+102 |  |  |  |  |  | x | x | | |
| KQ5+6 | 3 | 58+60+80 |  |  |  |  |  | x | x | | |
| KQ5+7 | 5 | 58+60+96+97+102 |  |  |  |  |  | x | x | | |
| KQ6+7 | 4 | 80+96+97+102 |  |  |  |  |  | x | x | | |

**Supplementary Table 2. Regression analysis of the de novo aggregation using various models.** The values correspond to fitted parameters of models shown in Figure 2 with error estimates obtained from the statsmodel module of python. AIC and BIC correspond to Akaike information criterion and Bayesian information criterion, respectively. Asterisks denote levels of statistical significance: * p < 0.05; ** p < 0.01; *** p < 0.001, ns – not significant.

| **model** | ln(t0.5) ~ G | | | | ln(t0.5) ~ G + #muts | | | | ln(t0.5) ~ G + Variant | | | | ln(t0.5) ~ G + S + E | | | |
| --- | --- | --- | --- | --- | --- | --- | --- | --- | --- | --- | --- | --- | --- | --- | --- | --- |
| **R^2^** | 0.73 | | | | 0.74 | | | | 0.88 | | | | 0.92 | | | |
| **Adjusted R^2^** | 0.72 | | | | 0.72 | | | | 0.87 | | | | 0.91 | | | |
| **AIC** | 262 | | | | 263 | | | | 196 | | | | 165 | | | |
| **BIC** | 272 | | | | 276 | | | | 224 | | | | 199 | | | |
| **parameter** | **β ± SD** | | | **p** | **β ± SD** | | | **p** | **β ± SD** | | | **p** | **β ± SD** | | | **p** |
| Intercept | 5.16 | ± | 1.04 | *** | 5.37 | ± | 1.05 | *** | 4.86 | ± | 0.73 | *** | 5.11 | ± | 0.63 | *** |
| log[M]_0_ | -0.77 | ± | 0.25 | ** | -0.78 | ± | 0.25 | ** | -0.55 | ± | 0.17 | ** | -0.52 | ± | 0.15 | *** |
| Q^2^ | 0.04 | ± | 0.00 | *** | 0.04 | ± | 0.00 | *** | 0.04 | ± | 0.00 | *** | 0.03 | ± | 0.00 | *** |
| √I | -6.98 | ± | 0.95 | *** | -7.26 | ± | 0.98 | *** | -7.26 | ± | 0.68 | *** | -5.39 | ± | 0.66 | *** |
| KQ1 |  |  |  |  |  |  |  |  | -0.07 | ± | 0.26 | n.s. | -0.12 | ± | 0.20 | n.s. |
| KQ2 |  |  |  |  |  |  |  |  | 0.26 | ± | 0.23 | n.s. | 0.07 | ± | 0.18 | n.s. |
| KQ3 |  |  |  |  |  |  |  |  | -0.48 | ± | 0.23 | * | -0.04 | ± | 0.21 | n.s. |
| KQ4 |  |  |  |  |  |  |  |  | -1.20 | ± | 0.22 | *** | -0.61 | ± | 0.18 | *** |
| KQ5 |  |  |  |  |  |  |  |  | -0.25 | ± | 0.24 | n.s. | -0.25 | ± | 0.17 | n.s. |
| KQ6 |  |  |  |  |  |  |  |  | -1.44 | ± | 0.19 | *** | -0.70 | ± | 0.18 | *** |
| KQ7 |  |  |  |  |  |  |  |  | -1.10 | ± | 0.23 | * | -0.39 | ± | 0.22 | *** |
| #muts |  |  |  |  | -0.11 | ± | 0.09 | n.s. |  |  |  |  |  |  |  |  |
| β -citrate |  |  |  |  |  |  |  |  |  |  |  |  | -0.89 | ± | 0.18 | *** |
| β -tris |  |  |  |  |  |  |  |  |  |  |  |  | -0.77 | ± | 0.14 | *** |

**Supplementary Table 3. Results of kinetic, thermodynamic, and morphological analyses of WT and KQ fibrils.** The values of relative changes of elongation barrier (ΔΔG^ǂ^) between mutant and WT, fibril stability (ΔG), and m-values correspond to means ± SEM (n=3-4). The values of AFM derived heights correspond to means ± SD (n = 11-118, **Figure 3**).

|  |  | **Elongation** | **Stability** | | **AFM** |
| --- | --- | --- | --- | --- | --- |
| **aSyn** | **K-Q mutated residues** | **ΔΔG^ǂ^ (kJ/mol)** | **ΔG**  **(kJ/mol)** | **m-value (kJ/M/mol)** | **Fibril height (nm)** |
| **WT** | - | - | -30.7 ± 1.6 | 6.4 ± 1.4 | 7.3 ± 0.8 |
| **KQ1** | 6+10+12 | -0.4 ± 0.9 | -28.3 ± 0.7 | 3.6 ± 0.2 | 5.4 ± 1.5 |
| **KQ2** | 21+23 | 1.9 ± 2.2 | -25.8 ± 1.1 | 4.8 ± 0.4 | 5.3 ± 1.4 |
| **KQ3** | 32+34 | -0.4 ± 0.4 | -29.5 ± 1.1 | 4.9 ± 0.3 | 5.1 ± 0.8 |
| **KQ4** | 43+45 | -8.5 ± 1.7 | -35.0 ± 1.8 | 7.1 ± 1.7 | 5.4 ± 1.3 |
| **KQ5** | 58+60 | -3.7 ± 1.5 | -32.7 ± 0.6 | 4.7 ± 1.1 | 5.1 ± 0.8 |
| **KQ6** | 80 | -8.2 ± 3.6 | -32.0 ± 0.8 | 5.9 ± 0.4 | 7.9 ± 1.5 |
| **KQ7** | 96+97+102 | 0.9 ± 3.4 | -27.4 ± 1.0 | 7.5 ± 1.8 | 7.5 ± 1.0 |

**Supplementary Table 4. Changes of energy barriers of elongation (ΔΔG^ǂ^) of single-point mutant, single cluster and double cluster variants on WT – Fm seed.** The values correspond to means ± SEM from n number of measurements.

| Single-point mutant variants | | | | | | |
| --- | --- | --- | --- | --- | --- | --- |
| **aSyn** | | **K-Q mutated** | | **ΔΔG^ǂ^ (kJ/mol)** | | |
| K6Q | 6 | | 5.8 ± 0.2 | | n=2 |  |
| K10Q | 10 | | 3.0 ± 0.8 | | n=2 |  |
| K12Q | 12 | | 3.4 ± 0.4 | | n=3 |  |
| K21Q | 21 | | 4.2 ± 0.5 | | n=2 |  |
| K23Q | 23 | | 4.7 | | n=1 |  |
| K32Q | 32 | | 3.4 ± 0.3 | | n=3 |  |
| K34Q | 34 | | 4.0 ± 0.5 | | n=2 |  |
| K43Q | 43 | | 3.1 ± 0.6 | | n=3 |  |
| K45Q | 45 | | 4.0 ± 0.3 | | n=2 |  |
| K58Q | 58 | | 5.0 ± 0.5 | | n=3 |  |
| K60Q | 60 | | 4.9 ± 0.4 | | n=2 |  |
| K96Q | 96 | | 2.6 ± 0.8 | | n=2 |  |
| K97Q | 97 | | 3.6 ± 0.5 | | n=2 |  |
| K102Q | 102 | | 4.7 ± 1.0 | | n=3 |  |
| Single cluster mutant variants | | | | | | |
| KQ1 | 6+10+12 | | 3.3 ± 0.4 | | n=4 |  |
| KQ2 | 21+23 | | 3.1 ± 0.7 | | n=4 |  |
| KQ3 | 32+34 | | 3.2 ± 0.8 | | n=4 |  |
| KQ4 | 43+45 | | 2.1 ± 1.3 | | n=7 |  |
| KQ5 | 58+60 | | 2.9 ± 1.0 | | n=3 |  |
| KQ6 | 80 | | 4.6 ± 1.4 | | n=8 |  |
| KQ7 | 96+97+102 | | 1.8 ± 0.5 | | n=4 |  |
| Double cluster mutant variants | | | | | | |
| 1+2 | 6+10+12+21+23 | | 10.6 ± 0.2 | | n=2 |  |
| 1+3 | 6+10+12+32+34 | | 7.2 ± 1-8 | | n=2 |  |
| 1+4 | 6+10+12+43+45 | | 6.3 ± 1.0 | | n=2 |  |
| 1+5 | 6+10+12+58+60 | | 9.8 ± 2.1 | | n=2 |  |
| 1+6 | 6+10+12+80 | | 5.0 ± 0.5 | | n=2 |  |
| 1+7 | 6+10+12+96+97+102 | | 8.9 ± 0.4, | | n=2 |  |
| 2+3 | 21+23+34+35 | | 6.0 ± 0.1 | | n=2 |  |
| 2+4 | 21+23+43+45 | | 5.0 | | n=1 |  |
| 2+5 | 21+23+58+60 | | 8.9 ± 1.5 | | n=2 |  |
| 2+6 | 21+23+80 | | 4.4 ± 1.4 | | n=2 |  |
| 2+7 | 21+23+96+97+102 | | 3.0 | | n=1 |  |
| 3+5 | 34+35+58+60 | | 4.7 | | n=1 |  |
| 3+6 | 34+35+80 | | 3.3 | | n=1 |  |
| 3+7 | 34+35+96+97+102 | | 4.2 ± 0.5 | | n=2 |  |
| 4+5 | 43+45+58+60 | | 4.6 ± 0.9 | | n=2 |  |
| 4+6 | 43+45+80 | | 1.8 ± 0.5 | | n=2 |  |
| 4+7 | 43+45+96+97+102 | | 3.5 ± 1.5 | | n=2 |  |
| 5+6 | 58+60+80 | | 6.3 ± 1.2 | | n=2 |  |
| 5+7 | 58+60+96+97+102 | | 3.5 ± 0.7 | | n=2 |  |
| 6+7 | 80+96+97+102 | | 2.3 ± 0.3 | | n=2 |  |

**Supplementary Table 5. Half-time analysis of ThT kinetics at low pH in the presence of 1 nM WT-Fm or WT-Ri polymorph.** The half-times were obtained by fitting equation M1 to the raw kinetic data. Values correspond to the mean ± SD from a triplicate measurement. The relative half times were obtained as t_0.5_ (mut)/t_0.5_(WT) where t_0.5_ is the aggregation half-time. n.d. – not determined.

| **aSyn** | **WT - Fm** | | **WT - Ri** | |
| --- | --- | --- | --- | --- |
|  | **Half-time (h)** | **Relative half-time** | **Half-time (h)** | **Relative half-time** |
| Dataset 1 | | | | |
| WT | 42.3 ± 2.7 | 1 | 99.7 ± 0.0 | 1 |
| KQ1 | 119.9 ± 20.5 | 2.8 | n.d. | |
| KQ2 | n.d. | | 65.7 ± 7.4 | 0.7 |
| KQ3 | 100.8 ± 5.2 | 2.4 | 107.3 ± 20.4 | 1.1 |
| KQ4 | 61.6 ± 12.3 | 1.5 | 99.3 ± 5.0 | 1 |
| KQ5 | 83.4 ± 13.9 | 2 | n.d. | |
| KQ6 | 72.8 ± 13.6 | 1.7 | 123.8 ± 31.0 | 1.2 |
| KQ7 | 28.5 ± 4.3 | 0.7 | 44.6 ± 6.5 | 0.4 |
| Dataset 2 | | | | |
| WT | 26.3 ± 0.0 | 1 | 52.8 ± 0.0 | 1 |
| KQ1+2 | 135.8 ± 0.0 | 5.2 | 158.3 ± 14.7 | 3 |
| KQ1+3 | n.d. | | n.d. | |
| KQ1+4 | 49.4 ± 4.7 | 1.9 | 80.2 ± 4.7 | 1.5 |
| KQ1+5 | 94.5 ± 15.4 | 3.6 | n.d. | |
| KQ1+6 | 79.7 ± 5.4 | 3 | 118.1 ± 12.1 | 2.2 |
| KQ1+7 | 66.1 ± 24.3 | 2.5 | 63.9 ± 8.4 | 1.2 |
| KQ2+3 | 145.9 ± 19.0 | 5.5 | n.d. | |
| KQ2+5 | 139.8 ± 12.3 | 5.3 | n.d. | |
| KQ2+6 | 62.6 ± 2.3 | 2.4 | 122.1 ± 24.1 | 2.3 |
| KQ2+7 | 62.0 ± 6.0 | 2.4 | 84.4 ± 14.9 | 1.6 |
| KQ3+5 | n.d. | | n.d. | |
| KQ3+6 | 50.4 ± 5.8 | 1.9 | 74.3 ± 4.7 | 1.4 |
| KQ3+7 | 79.0 ± 22.3 | 3 | 113.4 ± 9.0 | 2.1 |
| KQ4+5 | 91.3 ± 22.6 | 3.5 | 123.9 ± 2.0 | 2.3 |
| KQ4+6 | 37.0 ± 5.1 | 1.4 | 61.7 ± 2.6 | 1.2 |
| KQ4+7 | 39.1 ± 3.6 | 1.5 | 43.5 ± 2.8 | 0.8 |
| KQ5+6 | 74.5 ± 5.4 | 2.8 | 94.9 ± 12.6 | 1.8 |
| KQ5+7 | 77.8 ± 4.5 | 3 | 127.0 ± 0.0 | 2.4 |
| KQ6+7 | 26.9 ± 0.9 | 1 | 26.1 ± 1.5 | 0.5 |

**Supplementary Table 6. Half-time analysis of seed amplification assay (SAA) with brain homogenates.** Values represent means and standard error of the mean (SEM) calculated from n measurements that gave signal within the time course of the experiment.

|  |  | **MSA1** | **MSA2** | **MSA3** | **PD1** | **PD2** | **PD3** | **CTRL1** | **CTRL2** | **CTRL3** | **NEG** |
| --- | --- | --- | --- | --- | --- | --- | --- | --- | --- | --- | --- |
| WT | Mean  SEM  n | 51.87 6.21 4 | 60.33 11.10 4 | 51.06 6.42 4 | 137.49 0.00 1 | 69.44 15.96 2 | 129.16 0.00 1 | – – 0 | – – 0 | – – 0 | – – 0 |
| KQ1 | Mean  SEM  n | 54.75 2.30 4 | 93.99 16.11 4 | 60.74 2.77 4 | 125.31 0.00 1 | 90.52 3.49 4 | 150.05 0.00 1 | – – 0 | – – 0 | – – 0 | – – 0 |
| KQ2 | Mean  SEM  n | 89.30 3.53 4 | 120.43 25.74 2 | 92.13 9.52 3 | – – 0 | 135.04 0.00 1 | – – 0 | – – 0 | – – 0 | – – 0 | – – 0 |
| KQ3 | Mean  SEM  n | 70.60 7.25 4 | 84.43 7.46 4 | 58.59 13.06 4 | – – 0 | 90.21 6.38 4 | 92.67 12.39 4 | – – 0 | – – 0 | – – 0 | – – 0 |
| KQ4 | Mean  SEM  n | 22.41 1.47 4 | 21.43 1.44 4 | 21.44 1.63 4 | 66.84 6.69 4 | 33.70 3.33 4 | 64.53 2.83 4 | – – 0 | – – 0 | – – 0 | – – 0 |
| KQ5 | Mean  SEM  n | 104.38 13.09 4 | 90.73 17.44 4 | 86.60 16.99 4 | 61.54 0.00 1 | 94.01 26.45 3 | 96.00 0.00 1 | 127.69 0.00 1 | 87.17 0.00 1 | 141.88 0.00 1 | – – 0 |
| KQ6 | Mean  SEM  n | 60.29 6.31 4 | 88.32 2.52 4 | 114.76 8.86 3 | 77.40 4.34 4 | 120.48 0.00 1 | – – 0 | 45.25 6.78 3 | – – 0 | – – 0 | – – 0 |
| KQ7 | Mean  SEM  n | 50.26 17.17 4 | 30.47 4.77 4 | 34.55 3.97 4 | 82.96 10.14 4 | 49.42 7.46 4 | 76.49 3.00 4 | 93.92 22.82 3 | 140.75 0.00 1 | – – 0 | 141.37 13.23 2 |

**Supplementary Table 7. Comparison regression analyses coefficients from the de novo aggregation assays** carried out in large and small scale**.** The values correspond to fitted parameters of models shown in Figure 2 with error estimated obtained from the statsmodel module of python. AIC and BIC correspond to Akaike information criterion and Bayesian information criterion, respectively. Asterisks denote levels of statistical significance: * p < 0.05; ** p < 0.01; *** p < 0.001, ns – not significant.

| **model** | ln(t0.5) ~ G + S + E | | | | ln(t0.5) ~ G + S + E | | | |
| --- | --- | --- | --- | --- | --- | --- | --- | --- |
| **dataset** | Large scale (96W) | | | | Small scale (384W) | | | |
| **R^2^** | 0.92 | | | | 0.74 | | | |
| **Adjusted R^2^** | 0.91 | | | | 0.72 | | | |
| **AIC** | 165 | | | | 412 | | | |
| **BIC** | 199 | | | | 453 | | | |
| **parameter** | **β ± SD** | | | **p** | **β ± SD** | | | **p** |
| Intercept | 5.11 | ± | 0.63 | *** | 3.96 | ± | 0.41 | *** |
| log[M]_0_ | -0.52 | ± | 0.15 | *** | -0.52 | ± | 0.08 | *** |
| Q^2^ | 0.03 | ± | 0.00 | *** | 0.03 | ± | 0.00 | *** |
| √I | -5.39 | ± | 0.66 | *** | -2.88 | ± | 0.55 | *** |
| KQ1 | -0.12 | ± | 0.20 | n.s. | -0.39 | ± | 0.22 | n.s. |
| KQ2 | 0.07 | ± | 0.18 | n.s. | -0.11 | ± | 0.20 | n.s. |
| KQ3 | -0.04 | ± | 0.21 | n.s. | -0.24 | ± | 0.22 | n.s. |
| KQ4 | -0.61 | ± | 0.18 | *** | -0.80 | ± | 0.19 | *** |
| KQ5 | -0.25 | ± | 0.17 | n.s. | -0.42 | ± | 0.18 | * |
| KQ6 | -0.70 | ± | 0.18 | *** | -0.74 | ± | 0.19 | *** |
| KQ7 | -0.39 | ± | 0.22 | *** | -0.65 | ± | 0.24 | ** |
| β -citrate | -0.89 | ± | 0.18 | *** | 0.23 | ± | 0.16 | n.s. |
| β -tris | -0.77 | ± | 0.14 | *** | 0.06 | ± | 0.15 | n.s. |

**Supplementary Table 8. In-silico analysis of fibril stability changes upon K-to-Q mutation using FoldX.**

|  | **K-to-Q ddG (kJ/mol)** | | | | | | | | | | | | | | |
| --- | --- | --- | --- | --- | --- | --- | --- | --- | --- | --- | --- | --- | --- | --- | --- |
| PDB code | **6** | **10** | **12** | **21** | **23** | **32** | **34** | **43** | **45** | **58** | **60** | **80** | **96** | **97** | **102** |
| **2n0a** |  |  |  |  |  | 1.2 | -0.5 | 0.0 | -0.1 | 5.8 | 1.3 | 2.3 | 1.8 | 1.8 |  |
| **6cu7** |  |  |  |  |  |  |  | 0.4 | 2.2 | 0.7 | 0.8 | 5.2 | 2.5 | 1.4 |  |
| **6cu8** |  |  |  |  |  |  |  | -0.6 | 0.7 | 2.1 | 0.8 | 1.3 |  |  |  |
| **6rt0** |  |  |  | 2.5 | 1.0 |  |  | 1.0 | 2.8 | 1.4 | 1.4 | 1.5 | 1.1 | 0.9 |  |
| **6rtb** |  | 0.7 | 1.7 | 0.2 | 1.4 |  |  | 0.3 | 2.0 |  |  | 1.6 |  |  |  |
| **6sst** |  |  |  | 5.2 | 0.7 |  |  | 0.1 | 2.7 | -0.4 | 3.9 | 1.3 | 1.1 |  |  |
| **6ssx** |  |  |  | 0.6 | 0.7 |  |  | 0.0 | 2.8 | 2.2 | 4.5 | 1.8 | 2.5 |  |  |
| **6xyo** |  |  |  | 0.8 | 0.9 | 1.1 | 3.2 | 0.2 | 2.3 | 5.0 | 4.0 | 6.0 | 3.1 | 1.8 |  |
| **6xyp** |  |  |  | 2.3 | 0.4 | -1.0 | 1.2 | 0.3 | 0.1 | 3.0 | 4.8 | 4.3 | 3.4 | 2.6 |  |
| **6xyq** |  |  |  | 2.1 | -0.1 | -0.9 | 1.0 | 1.5 | 0.3 | 3.4 | 4.8 | 3.2 | 3.7 | 1.2 |  |
| **7nca** |  |  |  |  |  |  |  | 0.2 | 0.8 | 2.6 | 1.5 | 0.5 | 2.0 | 1.3 |  |
| **7ncg** |  |  |  |  |  |  |  | 0.4 | 3.2 | 4.7 | 2.6 | 1.6 | 3.2 | 1.3 |  |
| **7nch** |  |  |  | 2.2 | 0.1 |  |  | 0.8 | 1.4 | 0.6 | 2.5 | 0.1 |  |  |  |
| **7nci** |  |  |  | 2.0 | 0.2 |  |  | 0.6 | 1.4 | 1.7 | 2.5 | 0.9 |  |  |  |
| **7ncj** |  |  |  | 2.3 | -0.2 |  |  | 0.7 | 2.4 | 1.9 | 2.1 | 0.5 | 3.9 | 1.4 |  |
| **7nck** |  |  |  |  |  |  |  | 0.1 | 1.7 | 4.5 | 1.7 | 7.1 | 3.6 | 2.4 |  |
| **7ozg** |  |  |  | 4.7 | 0.8 |  |  | 0.6 | 1.3 | -0.4 | 2.3 | 1.3 | -1.0 |  |  |
| **7ozh** |  |  |  |  |  |  |  | 1.4 | 2.8 | 2.1 | 1.5 | 1.1 |  |  |  |
| **7v47** |  |  |  |  |  |  |  | 0.0 | 3.5 | 2.7 | 1.1 | 2.3 | 2.9 |  |  |
| **7v48** |  |  |  |  |  |  |  | 1.3 | 1.5 | 4.7 | 0.5 | 2.6 | 3.0 | 2.4 |  |
| **7v49** |  |  |  |  |  |  |  | 1.6 | 0.9 | -4.5 | 1.6 | 4.7 |  |  |  |
| **7xjx** |  |  |  |  |  |  |  | 0.6 | 3.7 | 3.2 | 1.8 | 5.1 | 1.8 | 3.5 |  |
| **7xo0** |  |  |  |  |  |  |  | 1.3 | 1.3 | 2.8 | -0.4 | 3.2 | 1.2 | 1.1 |  |
| **7xo1** |  |  |  |  |  |  |  | 1.0 | 2.7 | 2.4 | -0.6 | 2.6 | 3.9 | 1.0 |  |
| **7xo2** |  |  |  |  |  |  |  | 0.8 | 1.2 | 2.8 | 4.4 | 3.0 | 3.9 | 1.0 |  |
| **7xo3** |  |  |  |  |  |  |  | 1.6 | 2.0 | 3.0 | 0.5 | 2.1 | 2.4 | 3.7 |  |
| **7yk2** |  |  |  |  |  |  |  | -0.1 | 3.2 | 3.3 | 2.6 | 3.7 | 2.0 | 3.6 |  |
| **7yk8** |  |  |  | 0.0 | 0.7 |  |  | 1.0 | 4.3 | 3.1 | 2.3 | 4.2 | 0.7 | 1.0 |  |
| **7yng** |  |  |  |  |  |  |  | -0.4 | 1.4 | 3.2 | 1.6 | 3.8 | 1.7 | 0.3 |  |
| **7ynr** |  |  |  |  |  |  |  | 0.6 | 2.3 | 3.3 | 2.4 | 3.6 | 1.4 | 1.7 |  |
| **7yns** |  |  |  |  |  |  |  | 2.9 | 1.4 | 2.2 | 0.6 | 3.5 | 2.6 | -0.1 |  |
| **7ynt** |  |  |  |  |  |  |  | 0.5 | 2.8 | 3.6 | 2.5 | 2.6 | 2.9 | -0.3 |  |
| **8cyr** |  |  |  | 0.1 | 1.5 |  |  | 0.2 | 2.9 | 1.4 | 0.8 | 1.6 | 7.1 | 1.6 |  |
| **8cys** |  |  |  | 0.3 | 2.4 |  |  | 0.5 | 3.2 | 1.2 | 0.7 | 3.0 | 2.5 | 1.7 |  |
| **8cyt** |  |  |  | 3.3 | 1.5 |  |  | -0.6 | 2.4 | 1.9 | 4.9 | 2.3 | 6.0 | 1.1 |  |
| **8cyv** |  |  |  | 0.5 | 1.2 |  |  | -0.3 | 1.6 | 2.4 | 2.6 | 2.3 | 2.7 | 0.7 |  |
| **8cyw** |  |  |  | 6.3 | 0.4 |  |  | 0.9 | 1.7 | 2.5 | 3.8 | 1.2 | -0.3 |  |  |
| **8cyx** |  |  |  | 1.5 | 0.9 |  |  | 0.2 | 1.6 | -0.8 | 0.9 | 1.6 | -0.3 |  |  |
| **8cyy** |  |  |  | 1.6 | 1.8 |  |  | 0.0 | 3.4 | 3.6 | 2.6 | 2.4 | 5.1 | 1.8 |  |
| **8cz0** |  |  |  | 1.3 | 1.3 |  |  | 0.7 | 4.0 | 0.9 | 2.6 | 3.3 | 4.8 | 1.7 |  |
| **8cz1** |  |  |  | 2.6 | 1.3 |  |  | 0.7 | 1.8 | 1.1 | 0.7 | 3.0 | 2.9 | 1.2 |  |
| **8cz2** |  |  |  | 1.9 | 0.2 |  |  | 0.7 | 3.1 | 1.8 | 0.3 | 1.7 | 2.6 | 1.4 |  |
| **8cz3** |  |  |  | 3.3 | 1.8 |  |  | 0.5 | -0.1 | 3.1 | 4.6 | 1.9 | 3.3 | 0.9 |  |
| **8cz6** |  |  |  | 1.1 | 1.8 |  |  | -0.3 | 2.8 | 1.6 | -0.3 | 2.2 | 2.0 | 1.4 |  |
| **8h03** |  |  |  |  |  |  |  | 0.9 | 0.9 | 2.0 | 0.6 | 1.9 | 1.6 |  |  |
| **8h04** |  |  |  |  |  |  |  | 0.7 | 2.4 | 2.0 | -0.1 | 2.9 | 3.3 | 2.6 |  |
| **8h05** |  |  |  |  |  |  |  | 2.0 | 1.4 | 1.5 | 2.2 | 5.9 |  |  |  |
| **n - resolved** | 0 | 1 | 1 | 24 | 24 | 4 | 4 | 47 | 47 | 46 | 46 | 47 | 40 | 33 | 0 |
| **% - resolved** | 0.0 | 0.0 | 0.0 | 0.5 | 0.5 | 0.1 | 0.1 | 1.0 | 1.0 | 1.0 | 1.0 | 1.0 | 0.9 | 0.7 | 0.0 |
| **mean** |  | 0.7 | 1.7 | 2.0 | 0.9 | 0.1 | 1.2 | 0.6 | 2.0 | 2.2 | 2.0 | 2.7 | 2.6 | 1.5 |  |
| **SD** | 0.0 | 0.0 | 0.0 | 1.6 | 0.7 | 1.1 | 1.3 | 0.7 | 1.1 | 1.7 | 1.5 | 1.5 | 1.6 | 0.9 | 0.0 |

## References

1. Sawaya, M. R., Hughes, M. P., Rodriguez, J. A., Riek, R. & Eisenberg, D. S. The expanding amyloid family: Structure, stability, function, and pathogenesis. Cell 184, 4857–4873 (2021).

2. Schymkowitz, J. Borg J., Stricher F., Nys R., Rousseau F., Serrano L. The FoldX web server: an online force field. Nucleic Acids Research 33, W382–W388 (2005)

3. Connor, J. P., Radford, S. E. & Brockwell, D. J. Structural and thermodynamic classification of amyloid polymorphs. Structure 33, 1793-1804.e3 (2025).
